# Identification of prognostic candidate signatures by systematically revealing transcriptome characteristics in lung adenocarcinoma with differing tumor microenvironment immune phenotypes

**DOI:** 10.18632/aging.204112

**Published:** 2022-06-07

**Authors:** Qiang Chen, Jiakang Ma, Xiaoyi Wang, Xiangqing Zhu

**Affiliations:** 1 Faculty of Animal Science and Technology, Yunnan Agricultural University, Kunming, China; 2 Department of Medical Oncology, The Second Affiliated Hospital of Zhengzhou University, Zhengzhou, China; 3 Basic Medical Laboratory, The 920th Hospital of Joint Logistics Support Force of PLA, Kunming, China

**Keywords:** lung adenocarcinoma, transcriptome, tumor microenvironment, prognosis, bioinformatics

## Abstract

Accumulated evidence shows that tumor microenvironment plays crucial roles in predicting clinical outcomes of lung adenocarcinoma (LUAD). The current study aimed to identify some potentially prognostic signatures by systematically revealing the transcriptome characteristics in LUADs with differing immune phenotypes. LUAD gene expression data were retrieved from the public TCGA and GEO databases, and the transcriptome characteristics were systematically revealed using a comprehensive bioinformatics method including single-sample gene set enrichment analysis, differentially expressed gene (DEG) analysis, protein and protein interaction (PPI) network construction, competitive endogenous RNA (ceRNA) network construction, weighted gene coexpression network analysis and prognostic model establishment. Finally, 1169 key DEGs associated with LUAD immune phenotype, including 88 immune DEGs, were excavated. Five essential and eight immune essential DEGs were separately identified by constructing two PPI networks based on the above DEGs. Totals of 1085 key DElncRNAs and 45 key DEmiRNAs were excavated and one ceRNA network consisting of 26 DEmRNAs, 3 DEmiRNAs and 57 DElncRNAs were established. The most significant gene coexpression module (cor=0.63 and *p*=3e-55) associated with LUAD immune phenotypes and three genes (*FGR*, *BTK*, *SPI1*) related to the immune cell infiltration were identified. Three robust prognostic signatures including a 9-lncRNA, an 8-lncRNA and an 8-mRNA were established. The areas under the curves of 5-year correlated with overall survival rate were separately 0.7319, 0.7228 and 0.713 in the receiver operating characteristic curve. The findings provide novel insights into the immunological mechanism in LUAD biology and in predicting the prognosis of LUAD patients.

## INTRODUCTION

Lung cancer (LC) is one of the most common malignant tumors worldwide [1]. In 2018, there were more than 2 million new cases, accounting for the incidence of about 11.6% of total diagnosed cancer cases [2]. Especially in countries or regions with larger tobacco production and consumption, the incidence of LC has been increasing rapidly [3]. For example, the annual growth percentage of LC cases is 2%-3% in recent years in China [3]. In the UK, the overall LC incidence rate has increased by 4%, and increased rapidly by 18% in females between 2003-2005 and 2012-2014 [4]. However, the overall survival (OS) rate of LC is very poor, and the 5-year OS rate is not more than 20% [1].

Non-small cell lung cancer (NSCLC) is the most common LC histological type, constituting about 90% of all LC cases [5]. Lung adenocarcinoma (LUAD) is the major NSCLC subtype, representing more than 50% of all NSCLC cases in recent years and causing more than six hundred thousand deaths worldwide each year [5, 6]. Currently, the prognosis and treatment of LUAD patients are assessed mainly based on the tumor node metastasis (TNM) staging system [4]. However, the clinical outcomes vary greatly among patients within the same TNM stage on account of the high heterogeneity in LUADs, and the predictive values obtained from the pathological characteristics are clinically limited in predicting the survival [7]. Recently, some molecular features implicated in LUAD have been uncovered, and a few genes have been also used to evaluate the prognosis as potential predictors and combat LUAD as drug targets, such as *epidermal growth factor receptor* (*EGFR*) gene and *tumor protein* 53 (*TP53*) gene [8, 9]. In recent years, the accumulated evidence indicates that the tumor microenvironment (TME) play a key role in tumor initiation and progression, and can better predict the clinical outcome and assess the therapeutic efficacy than the TNM system [5, 10]. Especially, the distinct immune landscape of tumor-infiltrating immune cells in the tumoural niche can lead to the different prognoses and treatment responses [11], which demonstrates that the immunophenotype can be used to estimate the prognosis as an independent component in the classification system [12]. Presently, the comprehensive studies on the immunological characteristics of LUAD are still lacking based on the large-scale gene expression profiles.

In the current study, a large number of LUAD-related gene expression profiles were retrieved from the public TCGA database, and the molecular features in LUAD with differing immunity were systematically analyzed using a comprehensive bioinformatics method, including the evaluation for the abundance of immune cells by single sample gene set enrichment analysis (ssGSEA), the screening of key differentially expressed gene (DEG) via differentially expressed gene analysis (DEGA), the investigation of key gene function by functional enrichment analysis, the identification of gene coexpression module by weighted gene coexpression network analysis (WGCNA), the elucidation of interactive relationships among genes via protein and protein interaction (PPI) network, the revelation of regulatory relationships among ceRNAs through competitive endogenous RNA network (ceRNA) and the prediction of prognostic model on the basis of univariate and multivariate Cox regression models. Finally, we systematically revealed the transcriptome characteristics of LUADs with differing immune phenotypes and built three robust prognostic signatures to predict the prognoses of LUAD patients.

## RESULTS

**Figure 1:**
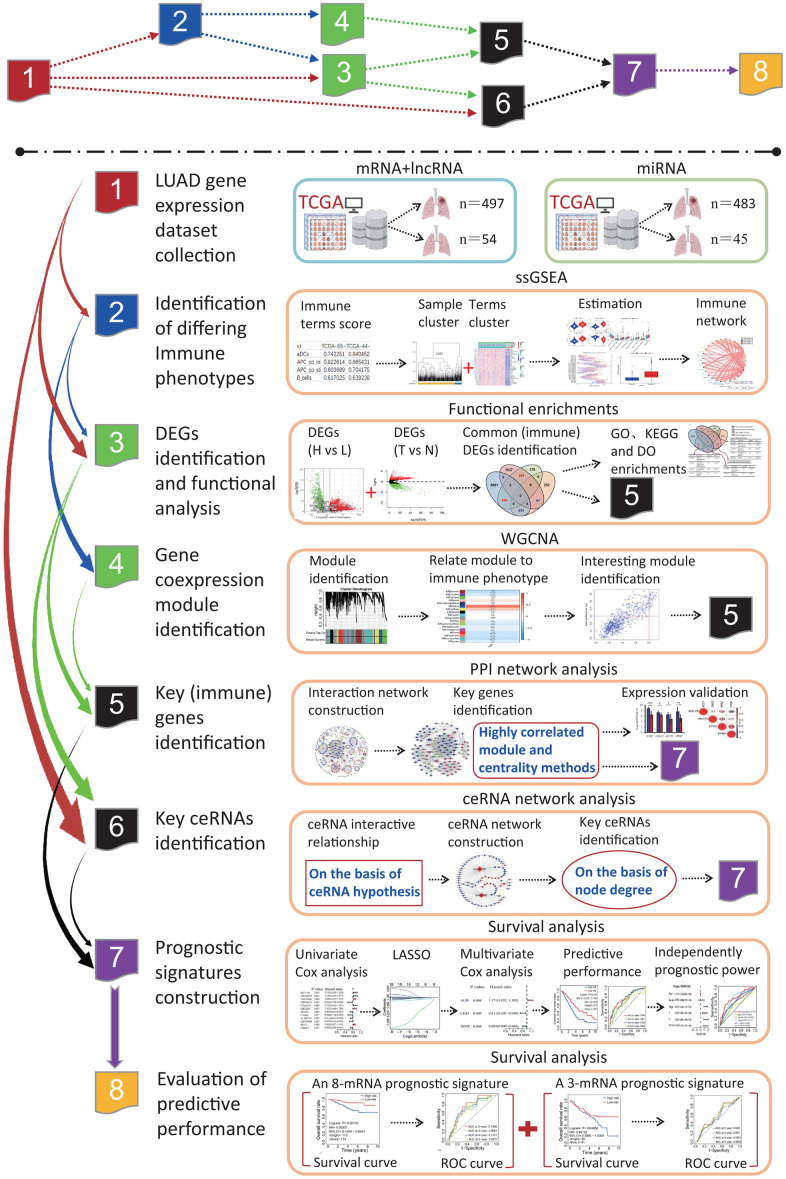
**The flow chart of systematic bioinformatics analysis.** In this study, a comprehensive bioinformatics method was used to reveal the transcriptome characteristics related to the LUADs with differing immune phenotypes and identify the prognostic signatures predicting the OS of LUAD patients, including ssGSEA, PPI network, WGCNA, ceRNA network, and survival analysis on the basis of univariate and multivariate Cox models. The predictive performances of two prognostic signatures were evaluated using two independent datasets. LUAD, lung adenocarcinoma; TCGA, the cancer genome atlas; ssGSEA, single-sample gene set enrichment analysis; PPI, protein and protein interaction; WGCNA, weighted gene coexpression network analysis; ceRNA, competitive endogenous RNA; GO, gene ontology; DO, disease ontology; KEGG, Kyoto Encyclopedia of Genes and Genomes; DEG, differentially expressed gene; LASSO, least absolute shrinkage and selection operator; OS, overall survival; ROC, receiver operating characteristic.

The flow chart of systematic bioinformatics analysis is displayed in Figure 1 in the current study. The basic steps are outlined as follows. (1) LUAD gene expression datasets including mRNA, lncRNA and miRNA and normal lung tissue samples were retrieved from the TCGA database. (2) LUAD patients were clustered into two subgroups or three subgroups according to the infiltration levels of immune cells using the ssGSEA method and the rationality of grouping patients was evaluated. (3) Two subgroups were separately the high and low immune infiltration subgroups and DEGs were identified between two immune infiltration subgroups. Further, DEGs were identified between the LUAD and normal lung tissues. The key DEGs were identified by an overlap analysis. (4) Three subgroups were separately the high, intermediate and low immune infiltration subgroups, and gene coexpression modules associated with immune phenotype were identified. (5) Three PPI networks were separately constructed on the basis of key DEGs, immune DEGs and genes in the most significant correlation module with immune phenotype. The essential genes were respectively identified using the molecular complex detection algorithm and centrality method in three PPI networks. (6) On the basis of the ceRNA hypothesis, one ceRNA network was constructed, and key ceRNAs were identified according to the degrees of all nodes in the ceRNA network. (7) The pivotal genes identified in each step were performed the survival analysis on the basis of univariate and multivariate Cox regression models. (8) The predictive performances of two prognostic signatures were evaluated using two independent datasets, respectively.

### Immune phenotype landscape in the TME of LUAD

**Figure 2:**
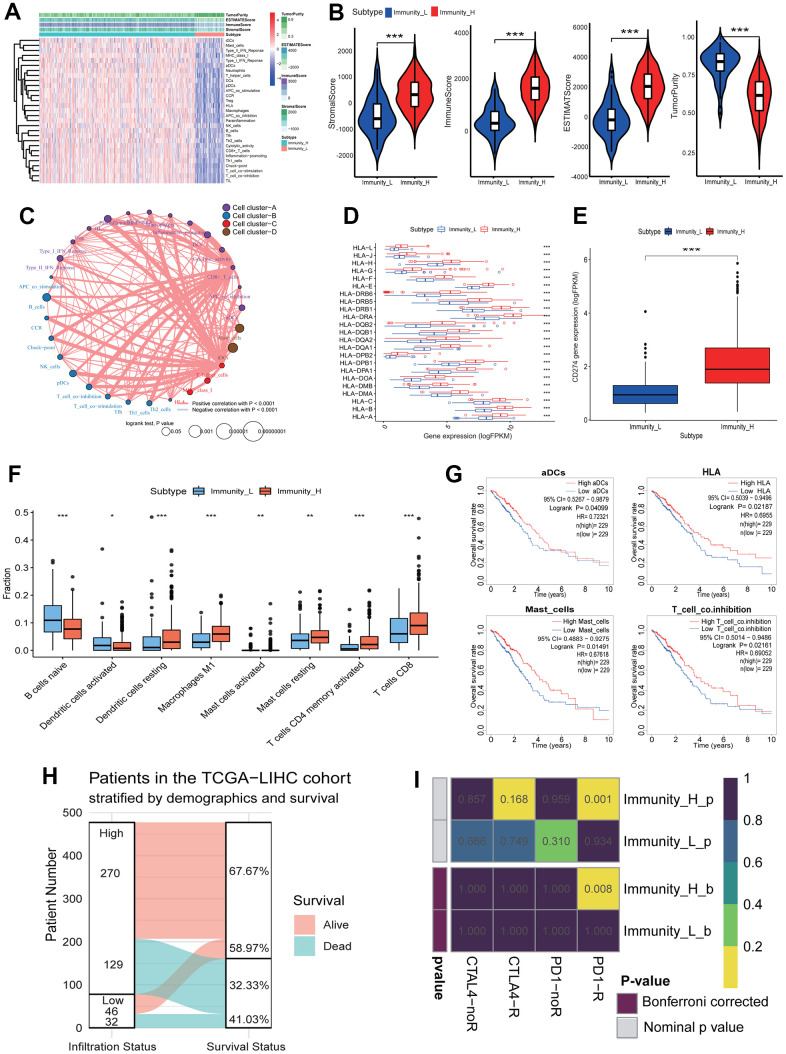
**Immune landscape of LUAD and the TME characteristics.** (**A**) Unsupervised clustering of LUAD patients from the TCGA cohort using ssGSEA scores from immune cell types. The “StromalScore” is the stromal signature that was designed to capture the presence of stroma in the tumor tissue. The “ImmuneScore” is the immune signature that aimed to represent the infiltration of immune cells in the tumor tissue. The “ESTIMATEScore” is the score combined by the stromal and immune scores. The “TumorPurity” is the tumor purity calculated by the nonlinear least squares method based on the ESTIMATEScore. The “Subtype” is the two clusters that were divided in the terms of the immune infiltration. The Immunity_H and Immunity_L subtypes showed the high and low immune infiltration, separately. (**B**) The ssGSEA scores in the differing TME immune phenotypes. The high immune infiltration group (Immunity_H) means the high StromalScore, ImmuneScore, ESTIMATEScore and low TumorPurity (all *p*<0.001). (**C**) Interaction of the TME immune cell types. The immune-related terms were clustered into 4 clusters according to the correlations among different immune-related terms. (**D**) The expressions of *HLA* genes in differing TME immune phenotypes. All *HLA* genes had significant differences in expression level between the high and low immune infiltration groups. (**E**) The expressions of *CD274* gene in differing TME immune phenotypes. The expression of *CD274* gene in the high immune infiltration group was significantly higher than that in the low immune infiltration group. (**F**) The fractions of the TME immune cells. The fractions of 8 immune cells had significant differences in two immune infiltration subgroups. (**G**) The associations of four immune cell types with overall survival. The high infiltration of aDCs, HLA, Mast_cells and T_cell_co.inhibition resulted in a higher OS of LUAD patients, respectively. (**H**) The relationships of immune infiltration status and survival status. The LUAD patients in the high immune infiltration group had a higher survival rate. (**I**) Responses of LUAD patients with differing TME immune phenotypes to immune therapy. The LUAD patients in the high immune infiltration group had significant response to anti-PD1-R. LUAD, lung adenocarcinoma; TME, tumor microenvironment; TCGA, the cancer genome atlas; ssGSEA, single-sample gene set enrichment analysis; OS, overall survival.

To assess the diverse immune responses in LUAD, the infiltration levels of 29 immune-related terms were assessed using the ssGSEA approach. The LUAD samples were divided into 2 immune infiltration subgroups (high immune infiltration: 418; low immune infiltration: 79) according to the immune infiltration (Figure 2A). The immune score, estimate score and stromal score in the high immune infiltration subgroup were significantly higher than those in the low immune infiltration subgroup (Kruskal-Wallis test, all *p*<0.001) (Figure 2B). Oppositely, the tumor purity score in the high immune infiltration subgroup was significantly lower than that in the low immune infiltration subgroup (Kruskal-Wallis test, *p*<0.001) (Figure 2B). This result indicates that the high infiltration subgroup has a higher proportion of immune and stromal cells, while the low infiltration subgroup has a higher proportion of tumor cells. Using the K-means cluster and hierarchical cluster methods, a 29-immune-related term network was constructed, depicting a comprehensive landscape of immune-related term interactions. The immune-related terms were clustered into 4 clusters in the immune-related term network, and the correlations were showed in Figure 2C among different immune-related terms. Notably, all *HLA* genes were significantly highly expressed in the high immune infiltration subgroup (unpaired t-test, all *p*<0.001) (Figure 2D). Moreover, the common immunotherapeutic target gene *CD274* (*PD-L1*) was also found to significantly highly expressed in the high infiltration subgroup (unpaired t-test, *p*<0.001) (Figure 2E). The comparison of immune cell subsets showed that dendritic cells resting (*p*<0.001), macrophages M1 (*p*<0.001), mast cells activated (*p*<0.01), mast cells resting (*p*<0.01), T cells CD4 memory activated (*p*<0.001) and T cells CD8 (*p*<0.001) had higher proportions in the high immune infiltration subgroup, while B cells naive (*p*<0.001) and dendritic cells activated (*p*<0.05) had lower proportions (Figure 2F). Survival analysis showed that aDCs (*p*=0.04099), HLA (*p*=0.02187), Mast_cell (*p*=0.01491) and T_cell_co.inhibition (*p*=0.02161) were significantly related to the overall survival (OS) of LUAD patients, and the higher immune score resulted in a higher OS rate (Figure 2G). Survival status showed that the alive patients in the high immune infiltration subgroup had a higher percentage than those in the low immune infiltration subgroup (67.67% vs 58.97%, Figure 2H).

To predict the clinical responses of LUAD patients with differing immune phenotypes to immune checkpoint blockade, we compared the expression profiles of LUAD patients that responded to immunotherapies between two immune infiltration subgroups. The result was observed that the patients in the high immune infiltration subgroup were more promising to respond to anti–PD-1 therapy (high immune infiltration subgroup: Bonferroni corrected *p*=0.008; low immune infiltration subgroup: Bonferroni corrected *p*=1.000) (Figure 2I), while the responds of patients to anti-CTLA4 therapy in two immune infiltration subgroups had no significant difference (both immune infiltration subgroups: Bonferroni corrected *p*=1.000).

### Gene alteration landscape of LUADs with differing TME immune phenotypes

**Figure 3:**
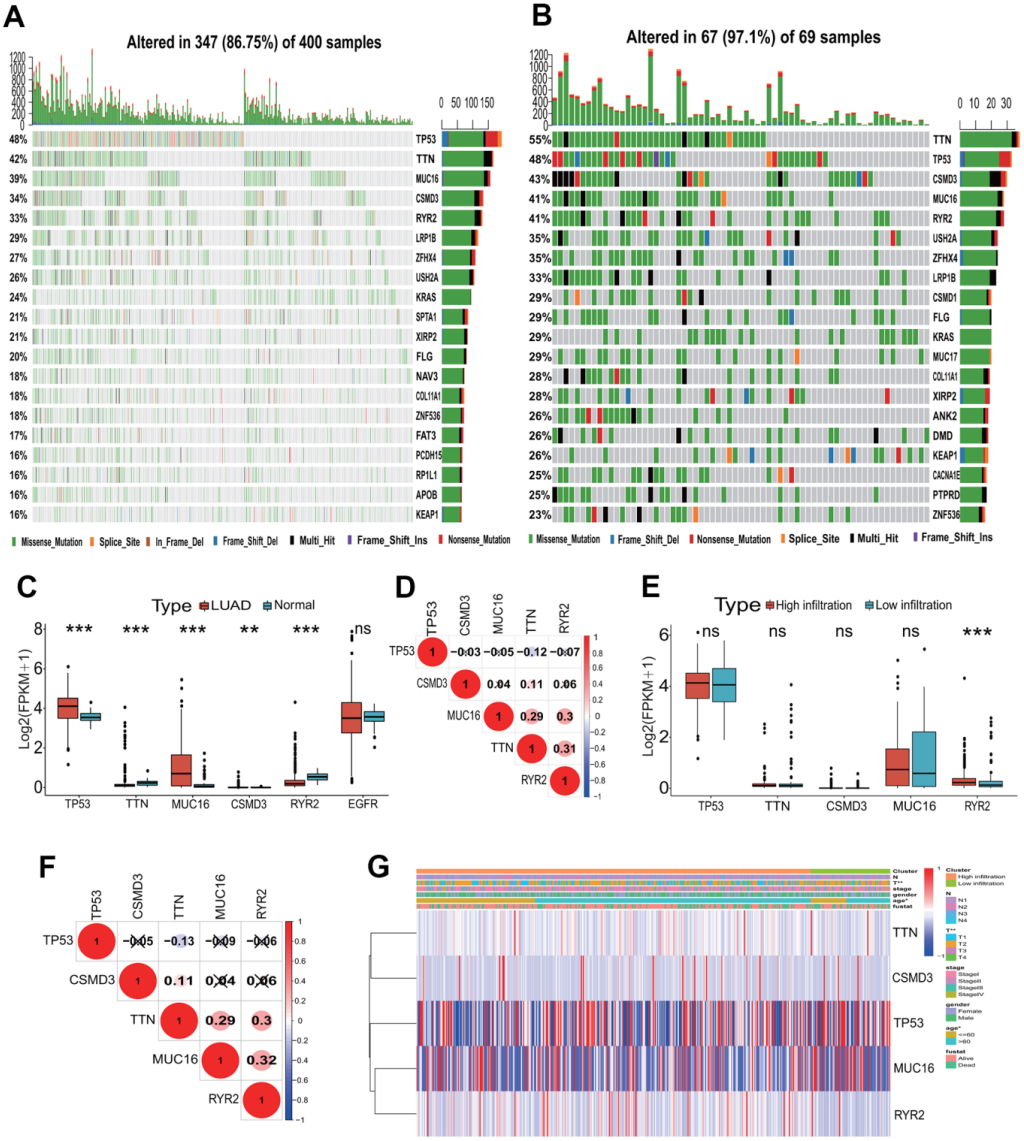
**Mutation landscape of the TME immune phenotype.** (**A**) Main mutant genes in the high immune infiltration group. *TP53*, *TTN*, *MUC16*, *CSMD3* and *RYR2* are the main mutant genes. (**B**) Main mutant genes in the low immune infiltration group. *TTN*, *TP53*, *CSMD3*, *MUC16* and *RYR2* are the main mutant genes. (**C**) The expressions of the top five mutant genes between LUAD and normal tissues. The expressions of five genes had significant differences between LUAD and normal tissues. (**D**) The expression correlations among the top five mutant genes. The correlations among the top five mutant genes were low in expressions. (**E**) The expressions of the top five mutant genes between the high and low immune infiltration groups. The expression of *RYR2* gene had significant difference between two groups. (**F**) The expression correlations of the top five mutant genes in two immune infiltration groups. The correlations among the top five mutant genes were low in expressions. (**G**) The associations of the top five mutant genes with clinical features in the TME immune phenotype. The expressions of these genes were significantly associated with T stage and age. TME, tumor microenvironment; LUAD, lung adenocarcinoma.

To investigate the gene alteration between the high and low immune infiltration subgroups, a gene alteration landscape was analyzed. The alteration landscapes of top 20 genes with higher alterations were showed in Figure 3A, 3B in two immune infiltration subgroups. Top 5 genes with the high alteration rate were *TP53*, *TTN*, *CSMD3*, *MUC16* and *RYR2* in two immune infiltration subgroups, and the missense mutation was the most important alteration type (Figure 3A, 3B). *TP53* and *TTN* ranked separately first in the high and low immune infiltration subgroups, constituting 48% and 55% of alteration rates. The 4 (*TP53*, *TTN*, *MUC16*, *CSMD3*) and 1 (*RYR2*) of 5 genes were significantly upregulated and downregulated in LUAD tissues (*p*<0.001 or 0.01, Figure 3C). There were lower correlations in expression among five genes between LUAD and normal lung tissues (Figure 3D). Except the significant high expression of *RYR2* gene (*p*<0.001), the expressions of the remaining four genes had no significant differences between two immune infiltration subgroups (Figure 3E). The expressions of five genes had lower correlations between two immune infiltration subgroups (Figure 3F). The expressions of five genes were significantly related to the age (*p*<0.05) and T stage (*p*<0.01) (Figure 3G).

### Key differentially expressed gene screening in LUADs with differing TME immune phenotypes and functional analysis

**Figure 4:**
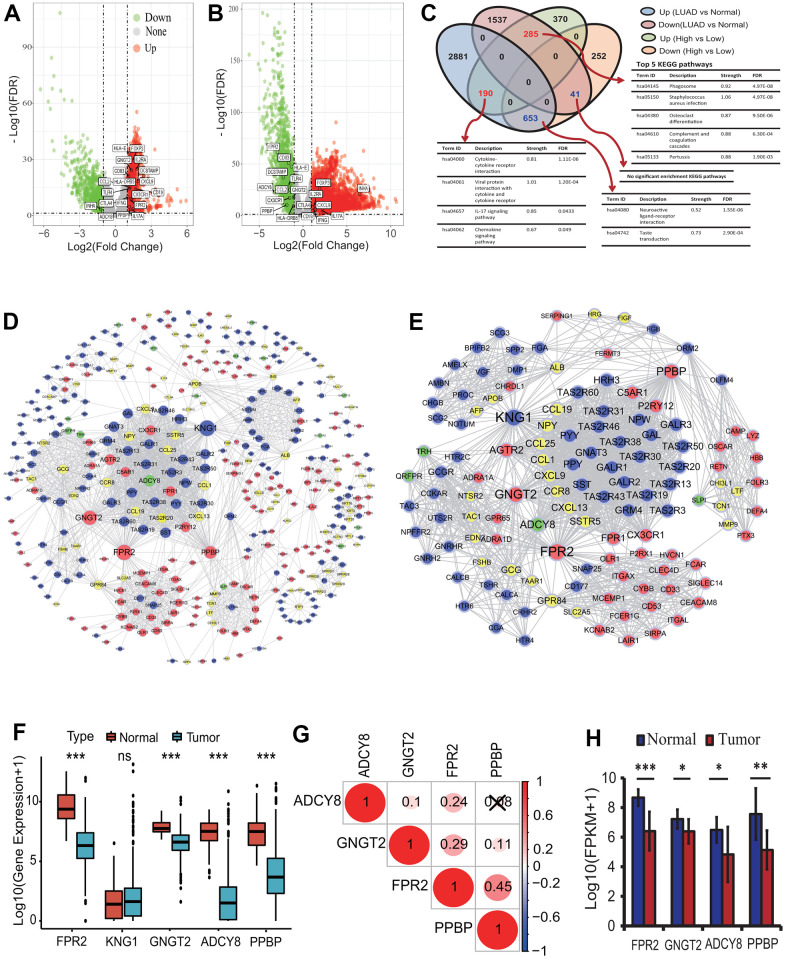
**Differentially expressed gene identification and analysis.** (**A**) The distribution of differentially expressed mRNAs between the high and low infiltration groups. Totals of 1791 DEmRNAs (845 upregulated and 946 downregulated) were identified according to the |LogFC|>1 and *p*<0.05. (**B**) The distribution of differentially expressed mRNAs between the LUAD and normal tissues. Totals of 5587 DEmRNAs (3724 upregulated and 1863 downregulated) were identified according to the |LogFC|>1 and *p*<0.05. (**C**) Key DEmRNAs identification and KEGG enrichment analysis. 1169 DEmRNAs were common in two types of DEmRNA sets from the comparison between the high and low infiltration groups and between the LUAD and normal lung tissues. These gene mainly involved in some immune KEGG pathways including cytokine-cytokine receptor interaction and IL-17 signaling pathway. (**D**) PPI network construction of key genes encoding proteins. A PPI network with 465 nodes and 2152 edges was established. (**E**) Identification of highly corelated module with the highest score. One module with 123 nodes and 1573 edges was identified. (**F**) The expression analysis of five genes between the LUAD and normal lung tissues. The expressions of four genes including *FPR2*, *GNGT2*, *ADCY8* and *PPBP* were significantly different between the LUAD and normal tissues. (**G**) The expression correlations among four essential genes. There were lower correlations in expression among four genes. (**H**) The expression analysis of four genes based on real transcriptome data. The expressions of four genes had significant differences between the LUAD and normal tissues. LUAD, lung adenocarcinoma; KEGG, Kyoto Encyclopedia of Genes and Genomes; DEG, differentially expressed gene; PPI, protein and protein interaction; FPKM, fragments per kilobase of exon model per million mapped fragments.

To identify key genes between the high and low immune infiltration subgroups, a DEGA was implemented. According to the statistical significance thresholds of |LogFC|>1 and *p*<0.05, totals of 1791 DEmRNAs including 845 upregulated and 946 downregulated were identified between the high and low immune infiltration subgroups (Figure 4A). Further, 5587 DEmRNAs consisting of 3724 upregulated and 1863 downregulated were identified between the LUAD tissues and normal lung tissues (Figure 4B). An overlap analysis showed that totals of 1169 DEmRNAs were common and were identified as key genes related to the immunophenotype of LUAD (Figure 4C and Supplementary Table 1). Among these common DEmRNAs, 190 DEmRNAs were significantly upregulated in the high infiltration subgroup and in the LUAD tissue. 653 DEmRNAs were significantly downregulated in the high infiltration subgroup and upregulated in the LUAD tissue. 285 DEmRNAs were significantly upregulated in the high infiltration subgroup and downregulated in the LUAD tissue, and 41 DEmRNAs were significantly downregulated in the high infiltration subgroup and in the LUAD tissue (Figure 4C). These genes were mainly associated with some immune KEGG pathways, such as cytokine-cytokine receptor interaction (Term ID: hsa04060), IL-17 signaling pathway (Term ID: hsa04657) and chemokine signaling pathway (Term ID: hsa04062) (Figure 4C).

To further understand the functions of the 1169 DEGs, a comprehensive functional analysis was performed. GO analysis showed that the 475 upregulated DEGs in the high immune infiltration group were significantly enriched within 112 GO BP terms (adjusted *p*<0.0001), and the top 5 BPs were separately neutrophil activation involved in immune response (GO:0002283, adjusted *p*=1.49E-17), neutrophil activation (GO:0042119, adjusted *p*=1.49E-17), neutrophil mediated immunity (GO:0002446, adjusted *p*=1.49E-17), neutrophil degranulation (GO:0043312, adjusted *p*=1.82E-17) and regulation of inflammatory response (GO:0050727, adjusted *p*=3.90E-11) (Table 1). Five KEGG pathways were significantly enriched, and the 5 KEGG pathways were separately staphylococcus aureus infection (Term ID: hsa05150, adjusted *p*=3.97E-06), viral protein interaction with cytokine and cytokine receptor (Term ID: hsa04061, adjusted *p*=3.97E-06), cytokine-cytokine receptor interaction (Term ID: hsa04060, adjusted *p*=3.97E-06), chemokine signaling pathway (Term ID: hsa04062, adjusted *p*=5.98E-05) and osteoclast differentiation (Term ID: hsa04380, adjusted *p*=5.98E-05) (Table 1). Twenty-one DO terms were significantly enriched, and the top 5 DO terms were separately skin disease (DO ID:37, adjusted *p*=6.01E-10), integumentary system disease (DO ID:16, adjusted *p*=3.30E-09), lung disease (DO ID:850, adjusted *p*=6.39E-09), dermatitis (DO ID:2723, adjusted *p*=6.39E-09) and chronic obstructive pulmonary disease (DO ID:3083, adjusted *p*=3.51E-08) (Table 1).

**Table 1:** Functional analysis of DEmRNAs encoding proteins.

**ID**	**Description**	**GeneRatio**	**pvalue**	**p.adjust**
**Upregulated GO BP terms**				
GO:0002283	neutrophil activation involved in immune response	52/416	4.39E-21	1.49E-17
GO:0042119	neutrophil activation	52/416	1.10E-20	1.49E-17
GO:0002446	neutrophil mediated immunity	52/416	1.20E-20	1.49E-17
GO:0043312	neutrophil degranulation	51/416	1.95E-20	1.82E-17
GO:0050727	regulation of inflammatory response	42/416	5.23E-14	3.90E-11
**Upregulated KEGG pathways**				
hsa05150	staphylococcus aureus infection	16/242	2.06E-08	3.97E-06
hsa04061	viral protein interaction with cytokine and cytokine receptor	16/242	3.76E-08	3.97E-06
hsa04060	cytokine-cytokine receptor interaction	28/242	4.90E-08	3.97E-06
hsa04062	chemokine signaling pathway	20/242	1.11E-06	5.98E-05
hsa04380	osteoclast differentiation	16/242	1.23E-06	5.98E-05
**Upregulated DO terms**				
DOID:37	skin disease	40/255	9.02E-13	6.01E-10
DOID:16	integumentary system disease	41/255	9.91E-12	3.30E-09
DOID:850	lung disease	46/255	3.24E-11	6.39E-09
DOID:2723	dermatitis	27/255	3.84E-11	6.39E-09
DOID:3083	chronic obstructive pulmonary disease	28/255	2.64E-10	3.51E-08
**Downregulated GO BP terms**				
GO:0001580	detection of chemical stimulus involved in sensory perception of bitter taste	14/567	8.02E-12	2.76E-08
GO:0050913	sensory perception of bitter taste	14/567	3.40E-11	5.85E-08
GO:0050912	detection of chemical stimulus involved in sensory perception of taste	14/567	6.58E-11	7.55E-08
GO:0050909	sensory perception of taste	15/567	1.15E-09	9.95E-07
GO:0048663	neuron fate commitment	14/567	7.10E-09	4.89E-06
**Downregulated KEGG pathways**				
hsa04080	neuroactive ligand-receptor interaction	39/242	2.56E-13	4.95E-11
hsa04742	taste transduction	18/242	5.16E-11	4.98E-09

DEmRNA, differentially expressed mRNA; GO, gene ontology; BP, biological processes; KEGG, Kyoto Encyclopedia of Genes and Genomes; DO, disease ontology.

The 694 downregulated DEGs in the high immune infiltration group were significantly enriched within 5 BPs and 2 KEGG pathways (Table 1). The 5 BPs were separately detection of chemical stimulus involved in sensory perception of bitter taste (GO:0001580, adjusted *p*=2.76E-08), sensory perception of bitter taste (GO:0050913, adjusted *p*=5.85E-08), detection of chemical stimulus involved in sensory perception of taste (GO:0050912, adjusted *p*=7.55E-08), sensory perception of taste (GO:0050909, adjusted *p*=9.95E-07) and neuron fate commitment (GO:0048663, adjusted *p*=4.89E-06). The 2 KEGG pathways were separately neuroactive ligand-receptor interaction (Term ID: hsa04080, adjusted *p*=4.95E-11) and taste transduction (Term ID: hsa04742, adjusted *p*=4.98E-09). No DO terms were significantly enriched.

### PPI network construction and key gene identification in LUADs with differing immune phenotypes

To reveal the interactive relationships and identify key genes among the 1169 DEGs encoding proteins, a PPI network was constructed. At the highest confidence (0.900), 465 of 1169 DEGs had 2152 gene-gene interactions and a PPI network consisting of 465 nodes and 2152 edges was established (Figure 4D). One highly correlated module (score: 25.787) with 123 nodes and 1573 edges was extracted from the whole PPI network according to the topological features (Figure 4E). Seven types of centrality scores for each gene in the module were calculated, and in terms of the scores for all genes obtained by each centrality method the top 10 genes with the highest score were identified (Table 2). The intersections of the top 10 genes from 7 centrality methods were separately *FPR2*, *KNG1*, *GNGT2*, *ADCY8* and *PPBP*, and were identified as the essential genes associated with the LUAD immune phenotype. The five essential genes were primarily related to the G protein-coupled receptor signaling pathway (GO:0007186, strength:1.19, FDR:0.0192). Four genes including *FPR2*, *GNGT2*, *ADCY8* and *PPBP* were significantly downregulated in the LUAD tissues (all *p*<0.001) (Figure 4F), and had lower correlations in expression (Figure 4G). An expression analysis based on RNA-seq data from 20 transcriptomes of 10 LUAD patients showed that the 4 genes were significantly downregulated in the LUAD tissues (paired t-test *p*<0.05 or 0.01 or 0.001, Figure 4H), which verified the expression results of four genes obtained from TCGA RNA-seq data. The expressions of four genes were not significantly related to the OS of the LUAD patients.

**Table 2:** Top genes with high centrality scores by seven centrality methods.

**Rank**	**Subgragh**		**Eigenvector**		**Information**		**Betweenness**		**Closeness**		**Network**		**Degree**	
	**Gene**	**Score**	**Gene**	**Score**	**Gene**	**Score**	**Gene**	**Score**	**Gene**	**Score**	**Gene**	**Score**	**Gene**	**Score**
**Differentially expressed genes**														
1	FPR2	4.07E15	FPR2	0.1721	KNG1	16.8330	KNG1	3560.7246	KNG1	0.7349	KNG1	75.5865	KNG1	79
2	KNG1	4.05E15	KNG1	0.1716	FPR2	16.7879	FPR2	3009.4500	FPR2	0.7134	FPR2	74.9214	FPR2	77
3	GNGT2	3.99E15	GNGT2	0.1704	GNGT2	16.2239	PPBP	2487.2605	GNGT2	0.6703	GNGT2	64.7296	GNGT2	69
4	ADCY8	3.53E15	ADCY8	0.1603	PPBP	15.6456	GNGT2	1062.7612	PPBP	0.6524	PPBP	51.4998	PPBP	57
5	AGTR2	3.47E15	AGTR2	0.1590	ADCY8	15.1808	ORM2	670.4247	ADCY8	0.6193	ADCY8	48.3857	ADCY8	51
6	PPBP	3.44E15	PPBP	0.1582	AGTR2	14.6327	ADCY8	609.8803	AGTR2	0.6010	AGTR2	41.0793	AGTR2	45
7	SST	3.37E15	SST	0.1566	SST	14.2092	GPR84	460.3634	SST	0.5894	SST	39.2799	FPR1	43
8	PPY	3.33E15	PPY	0.1558	FPR1	14.2092	FGA	402.2432	FPR1	0.5894	PPY	39.0839	SST	42
9	GNAT3	3.33E15	GNAT3	0.1558	PPY	14.0947	ITGAX	304.3922	PPY	0.5865	GNAT3	39.0839	GNAT3	42
10	PYY	3.33E15	PYY	0.1558	GNAT3	14.0947	CYBB	279.9222	GNAT3	0.5865	PYY	39.0839	NPY	41
**Differentially expressed immune genes**														
1	IL17A	1.64E08	IL17A	0.2518	IL17A	12.4557	IL17A	94.7455	IL17A	0.9487	IL17A	33.8295	IL17A	35
2	FOXP3	1.45E08	FOXP3	0.2373	FOXP3	12.0953	FOXP3	67.1044	FOXP3	0.8810	FOXP3	29.4223	FOXP3	32
3	CTLA4	1.33E08	CTLA4	0.2273	CTLA4	11.8302	CTLA4	49.8940	CTLA4	0.8410	CTLA4	26.9259	CTLA4	30
4	TLR4	1.31E08	TLR4	0.2251	TLR4	11.6894	TLR4	38.2315	TLR4	0.8222	TLR4	25.5208	TLR4	29
5	IFNG	1.26E08	IFNG	0.2206	IFNG	11.5425	IFNG	32.8076	IFNG	0.8044	IFNG	24.2196	IFNG	28
6	CCL2	1.18E08	CCL2	0.2134	CCL2	11.5425	CCL2	40.9951	CCL2	0.8044	CCL2	24.2021	CCL2	28
7	CD19	1.16E08	CD19	0.2120	CD19	11.3891	CD19	26.8774	CD19	0.7872	CD19	23.2694	CD19	27
8	CXCL9	1.09E08	CXCL9	0.2056	CXCL9	11.3891	CXCL9	40.0456	CXCL9	0.7872	CXCL9	22.6396	CXCL9	27

### Relationship between DEGs and the overall survival of LUAD patients

**Figure 5:**
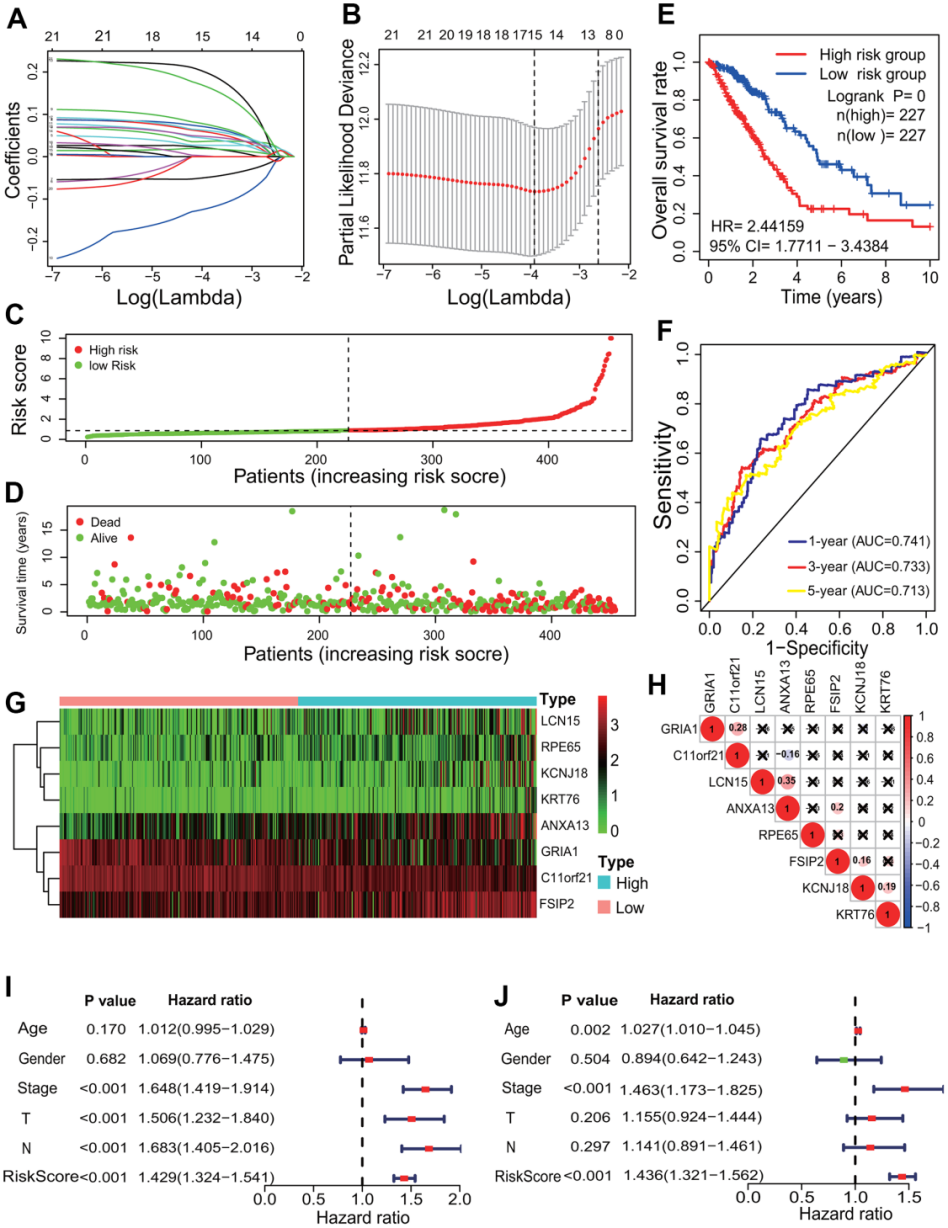
**Prognostic signature analysis of key differentially expressed genes.** (**A**, **B**) LASSO Cox analysis. Fifteen DEGs most correlated with the overall survival were identified, and 10-round cross validation was performed to prevent overfitting. (**C**) Risk score distribution. The LUAD patients were divided into the high- and low-risk groups according to the median risk score. (**D**) Survival overview. The distribution of survival times of the LUAD patients in the high- and low-risk groups. (**E**) Survival curve. The patients in the low-risk group exhibited a better overall survival rate than those in the high-risk group (*p*=0, HR=2.44159, 95% CI=1.7711-3.4384). (**F**) ROC curve. The ROC curve showed that the AUCs of 1-, 3- and 5-year of the 8-mRNA prognostic signature were separately 0.741, 0.733 and 0.713. (**G**) The heatmap of gene expression. Six DEGs (*KCNJ18*, *RPE65*, *LCN15*, *ANXA13*, *FSIP2* and *KRT76*) and two DEGs (*GRIA1*, *C11orf21*) were highly and lowly expressed in the high-risk group, respectively. (**H**) Expression correlation among genes. The expressions among 8 DEGs had no significant correlations. (**I**, **J**) Independent prognostic analysis. The 8-mRNA prognostic signature was significantly correlated with the OS of LUAD patients by a univariate Cox regression analysis and a multivariate Cox regression analysis. LASSO, least absolute shrinkage and selection operator; LUAD, lung adenocarcinoma; ROC, receiver operating characteristic; AUC, area under curve; OS, overall survival, DEG, differentially expressed gene.

To identify the relationships between the 1169 DEGs and the survival of LUAD patients, the survival analysis and prognostic model were performed. According to the *p*<0.001, 21 of the 1169 DEGs had significantly prognostic values by a univariate regression analysis (Supplementary Table 2). A lasso regression analysis showed that 15 of the 21 DEGs had the most important features in predicting the survival of LUAD patients (Figure 5A, 5B). Furthermore, 8 of the 15 DEGs (*KCNJ18*, *RPE65*, *GRIA1*, *LCN15*, *C11orf21*, *ANXA13*, *FSIP2* and *KRT76*) had significantly prognostic values by a multivariate Cox regression analysis. The risk scores for all LUAD patients were calculated, and according to the median risk score the patients were divided into the high- and low-risk groups (Figure 5C, 5D). The Kaplan-Meier curve showed that the LUAD patients in the high-risk group had a poorer OS rate (LR *p*=0, HR=2.4416, 95%CI=1.7711-3.4384) (Figure 5E). The AUCs of 1-, 3-, and 5-year associated with the OS were separately 0.741, 0.733 and 0.713 in the ROC curve (Figure 5F), which indicates a higher reliability of the 8-mRNA signature in predicting the survival. In the 8-mRNA prognostic signature, 6 DEGs (*KCNJ18*, *RPE65*, *LCN15*, *ANXA13*, *FSIP2* and *KRT76*) were significantly upregulated and 2 DEGs (*GRIA1*, *C11orf21*) were significantly downregulated in the high-risk group (Figure 5G). The expression correlations among the 8 DEGs were very low (Figure 5H). An independent prognostic analysis showed that the risk score of 8-mRNA prognostic signature had a significant association with the survival of LUAD patients by a univariate (*p*<0.001, HR=1.429, 95%CI=1.324-1.541) and a multivariate (*p*<0.001, HR=1.436, 95%CI=1.321-1.562) Cox regression analyses (Figure 5I, 5J).

### Immune DEGs identification and interactive relationship network construction

Among the identified 1169 DEGs, 88 DEGs (80 upregulated and 8 downregulated) are the genes related to the immunity (Supplementary Table 3). Eighty upregulated immune DEGs were mainly associated with immune response and immune regulation (Supplementary Table 4), and mainly have the molecular functions of chemokine, cytokine, chemokine receptor, cytokine receptor binding and activity. Eight downregulated immune DEGs mainly have molecular functions of hormone and receptor ligand activity.

**Figure 6:**
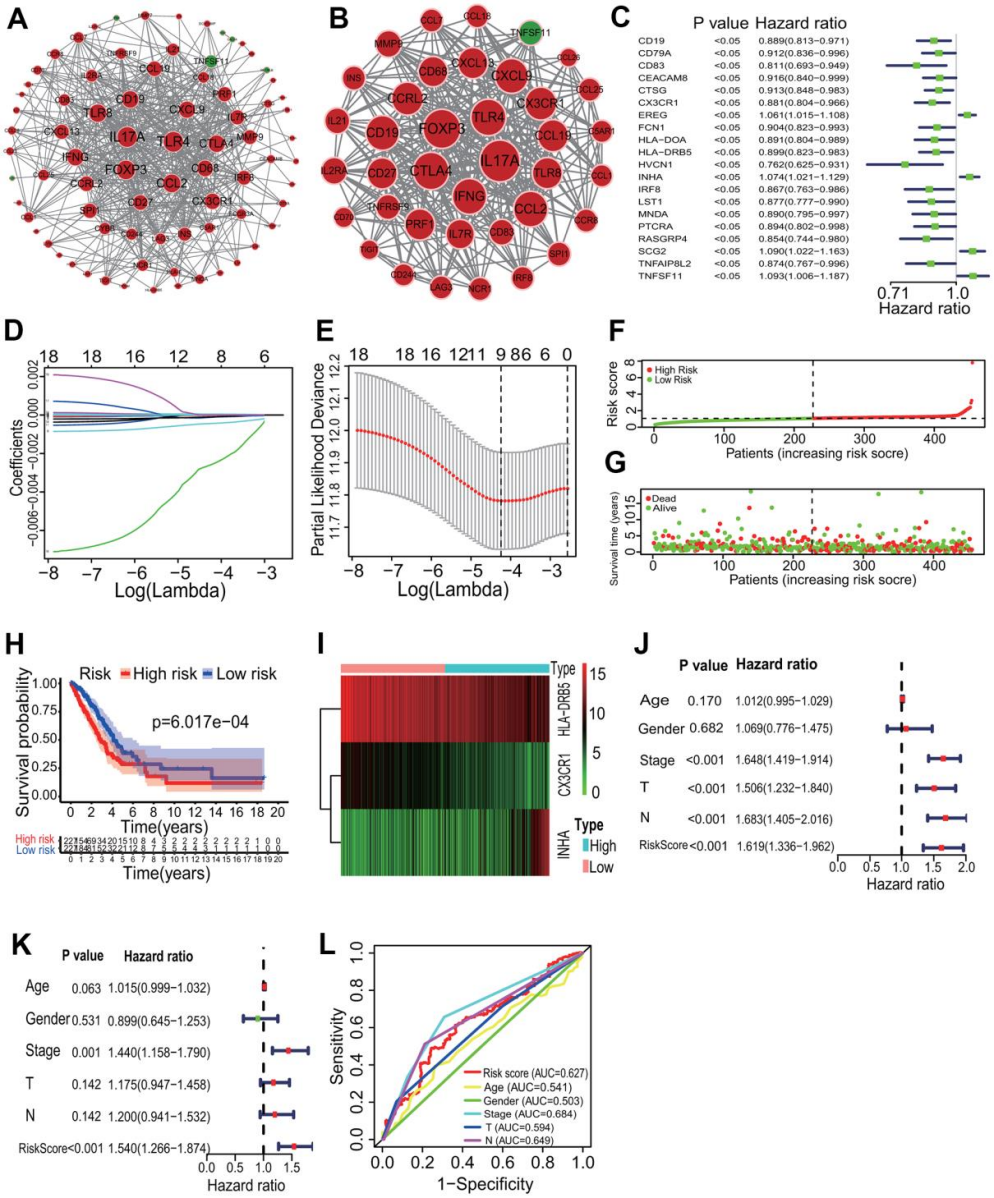
**Key immune gene identification and prognostic immune signature construction.** (**A**) PPI network of immune DEGs. An immune PPI network with 79 nodes and 538 edges was established. (**B**) Highly correlated PPI network. An immune PPI subnetwork with 38 nodes and 372 edges was constructed in the whole immune PPI network. (**C**) Univariate regression analysis. Twenty immune genes had significantly prognostic values. (**D**, **E**) LASSO Cox analysis. Nine immune genes most correlated with the overall survival were identified, and 10-round cross validation was performed to prevent overfitting. (**F**) Risk score distribution. LUAD patients were divided into the high- and low-risk groups according to the median risk score. (**G**) Survival overview. The distribution of survival times of LUAD patients in the high- and low-risk groups. (**H**) Survival curve. A better overall survival of patients in the low-risk group was exhibited than that in the high-risk group. (**I**) Heatmap of gene expression. *HLA-DRB5* and *CX3CR1* were highly expressed and *INHA* was lowly expressed in the high-risk group. (**J**, **K**) Independent prognostic analysis. The 3-mRNA risk signature was significantly correlated with the OS of LUAD patients by a univariate and a multivariate Cox regression analysis. (**L**) ROC curve. The AUC of 3-year survival was 0.627. PPI, protein and protein interaction; LASSO, least absolute shrinkage and selection operator; LUAD, lung adenocarcinoma; ROC, receiver operating characteristic; AUC, area under the curve; OS, overall survival.

The interactive relationships showed that 79 of the 88 immune DEGs had 538 gene-gene interaction pairs at the highest confidence 0.900, and an immune PPI network consisting of 79 nodes and 538 edges was established (Figure 6A). In accordance with the topological properties of whole immune PPI network, two highly correlated modules were identified using the MCODE algorithm, and the module with the higher score included 38 nodes and 372 edges (score=20.108, Figure 6B). A centrality analysis showed that 8 genes (*IL17A*, *FOXP3*, *CTLA4*, *TLR4*, *IFNG*, *CCL2*, *CD19*, *CXCL9*) had higher comprehensive centrality scores (Table 2), and were identified as essential immune genes in LUADs with differing TME immune phenotypes.

### Immune prognostic signature construction

According to the *p*<0.05, 20 immune DEGs had significantly prognostic values by a univariate regression analysis (Figure 6C). Among, 9 immune DEGs had the most important features in predicting the survival of LUAD patients by the Lasso regression analysis (Figure 6D, 6E). The multivariate Cox regression analysis showed that 3 (*HLA-DRB5*, *CX3CR1* and *INHA*) of the 9 immune DEGs had significant prognostic values. In the light of the median risk score, the patients were divided into the high- and low-risk groups (Figure 6F, 6G). In the low-risk group, LUAD patients had a higher OS rate in the Kaplan-Meier curve (LR *p*=6.017e-04) (Figure 6H). In the immune prognostic signature, *HLA-DRB5* and *CX3CR1* had lower expression and *INHA* had higher expression in the low-risk group (Figure 6I). An independently prognostic analysis showed that the risk score of 3-gene signature was significantly related to the survival of LUAD patients by a univariate (*p*<0.001, HR=1.619, 95%CI=1.336-1.962) and a multivariate (*p*<0.001, HR=1.540, 95%CI=1.266-1.874) Cox regression analyses (Figure 6J, 6K). The AUC of risk score was 0.627 in the ROC curve (Figure 6L).

### Identification of key DElncRNAs and DEmiRNAs related to LUAD immune phenotype and prognostic signature construction

**Figure 7:**
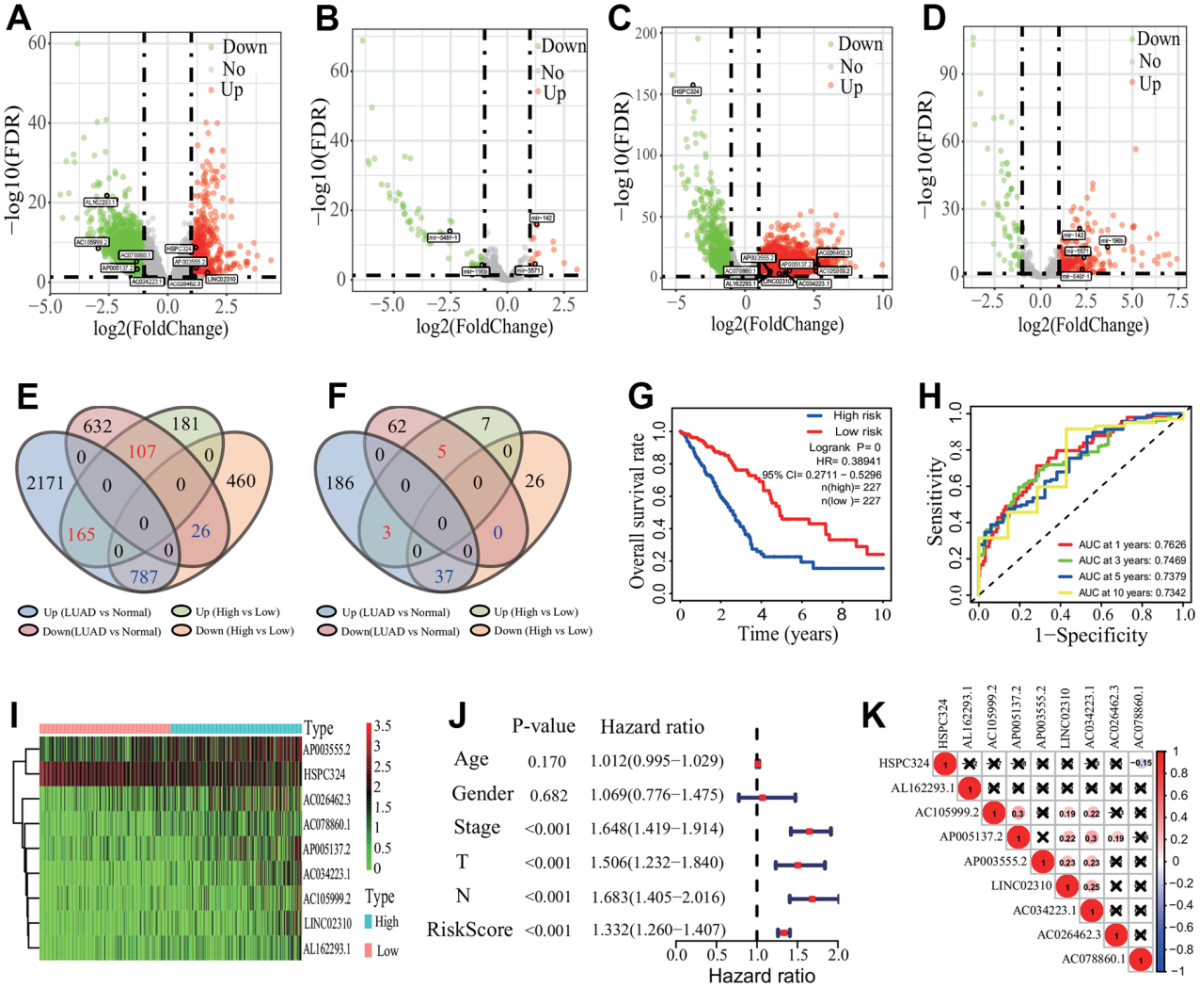
**Differentially expressed lncRNAs and miRNA identification and prognostic signature construction.** (**A**, **B**) Distribution of differentially expressed lncRNA and miRNA between the high and low immune infiltration groups. Totals of 1726 DElncRNAs and 78 DEmiRNAs were separately identified. (**C**, **D**) Distribution of differentially expressed lncRNA and miRNA between the LUAD and normal tissues. Totals of 3888 DElncRNAs and 293 DEmiRNAs were separately identified. (**E**, **F**) Key DElncRNA and DEmiRNA identification. Totals of 1085 key DElncRNAs and 45 key DEmiRNAs were respectively identified by an overlap analysis. (**G**) Survival curve. LUAD patients had a higher OS rate in the low-risk group (*p*=0, HR=0.38941, 95% CI=0.2711-0.5296). (**H**) ROC curve. The AUCs of 1-, 3, 5- and 10-years of 9-lncRNA signature were separately 0.7626, 0.7469, 0.7379 and 0.7342. (**I**) Heatmap of gene expression. Eight lncRNAs and one lncRNA were lowly and highly expressed in the low-risk group, respectively. (**J**) Independent prognostic analysis. The 9-lncRNA prognostic signature was significantly correlated with the OS of LUAD patients. (**K**) Expression correlation among 9 lncRNAs. The 9 lncRNAs had no significant correlations in expression. LUAD, lung adenocarcinoma; DElncRNA, differentially expressed lncRNA; DEmiRNA, differentially expressed miRNA; ROC, receiver operating characteristic; AUC, area under the curve; OS, overall survival, CI, confidence interval.

According to the |LogFC|>1 and *p*<0.05, 1726 DElncRNAs (453 upregulated and 1273 downregulated) and 78 DEmiRNAs (15 upregulated and 63 downregulated) were respectively identified between the high and low infiltration subgroups (Figure 7A, 7B). Further, 3888 DElncRNAs (3123 upregulated and 765 downregulated) and 293 DEmiRNAs (226 upregulated and 67 downregulated) were separately identified between the LUAD tissues and normal lung tissues (Figure 7C, 7D). An overlap analysis showed that totals of 1085 DElncRNAs and 45 DEmiRNAs were common (Figure 7E, 7F and Supplementary Table 1).

According to the *p*<0.001, 26 of the 1085 DElncRNAs had significantly prognostic values by a univariate regression analysis (Supplementary Table 2). A multivariate Cox regression analysis based on the 26 DElncRNAs showed that 9 DElncRNAs (*AP003555.2*, *LINC02310*, *AC026462.3*, *AL162293.1*, *AC078860.1*, *AC034223.1*, *HSPC324*, *AC105999.2* and *AP005137.2*) had significantly prognostic values. In accordance with the median risk score, the patients were divided into the high- and low-risk groups. The Kaplan-Meier curve showed that LUAD patients appeared a higher OS rate in the low-risk group (LR *p*=0, HR=0.3894, 95%CI=0.2711-0.5296) (Figure 7G). The AUCs of 1-, 3-, 5- and 10-year related to the OS were separately 0.7626, 0.7469, 0.7379 and 0.7342, which showed a higher effectiveness to predict the survival of LUAD patients (Figure 7H). In the 9-lncRNA prognostic signature, 8 DElncRNAs (*AP003555.2*, *LINC02310*, *AC026462.3*, *AL162293.1*, *AC078860.1*, *AC034223.1*, *AC105999.2* and *AP005137.2*) were lowly expressed and 1 lncRNA (*HSPC324*) was highly expressed in the low-risk group (Figure 7I). An independent prognostic analysis showed that the risk score of 9-lncRNA prognostic signature was significantly correlated with the survival of LUAD patients (*p*<0.001, HR=1.332, 95% CI=1.260-1.407, Figure 7J). The expression analysis showed the low expression correlations among the 9 DElncRNAs (Figure 7K).

Five (*mir-6850*, *mir-196b*, *mir-142*, *mir-548f-1*, *mir-5571*) of the 45 DEmiRNAs had significantly prognostic values according to the *p*<0.05 by a univariate regression analysis (Supplementary Table 2). A multivariate Cox regression analysis based on the 5 DEmiRNAs showed that 4 DEmiRNAs (*mir-196b*, *mir-142*, *mir-548f-1*, *mir-5571*) had significantly prognostic values, and the 3-year AUC of the 4-miRNA signature was 0.634 in the ROC curve (Supplementary Figure 1A). According to the median risk score, the patients were divided into the high- and low-risk groups (Supplementary Figure 1B, 1C). The Kaplan-Meier curve showed that LUAD patients had a higher HR and a lower OS in the high-risk group (LR *p*=0.00149, HR=1.6643, 95%CI=1.2147-2.3129) (Supplementary Figure 1D). The heatmap showed that 2 miRNAs (*mir-196b* and *mir-548f-1*) were highly expressed and 2 miRNAs (*mir-142* and *mir-5571*) were lowly expressed in the high-risk group (Supplementary Figure 1E). The expression correlations between the 4 miRNAs were low (Supplementary Figure 1F). An independently prognostic analysis showed that the 4-miRNA risk signature was significantly correlated with the survival of LUAD patients by a univariate (*p*<0.001, HR=1.531, 95%CI=1.344-1.744) and a multivariate (*p*<0.001, HR=1.439, 95%CI=1.237-1.674) Cox regression analyses (Supplementary Figure 1G, 1H).

### CeRNA network construction and prognostic ceRNA signature analysis

**Figure 8:**
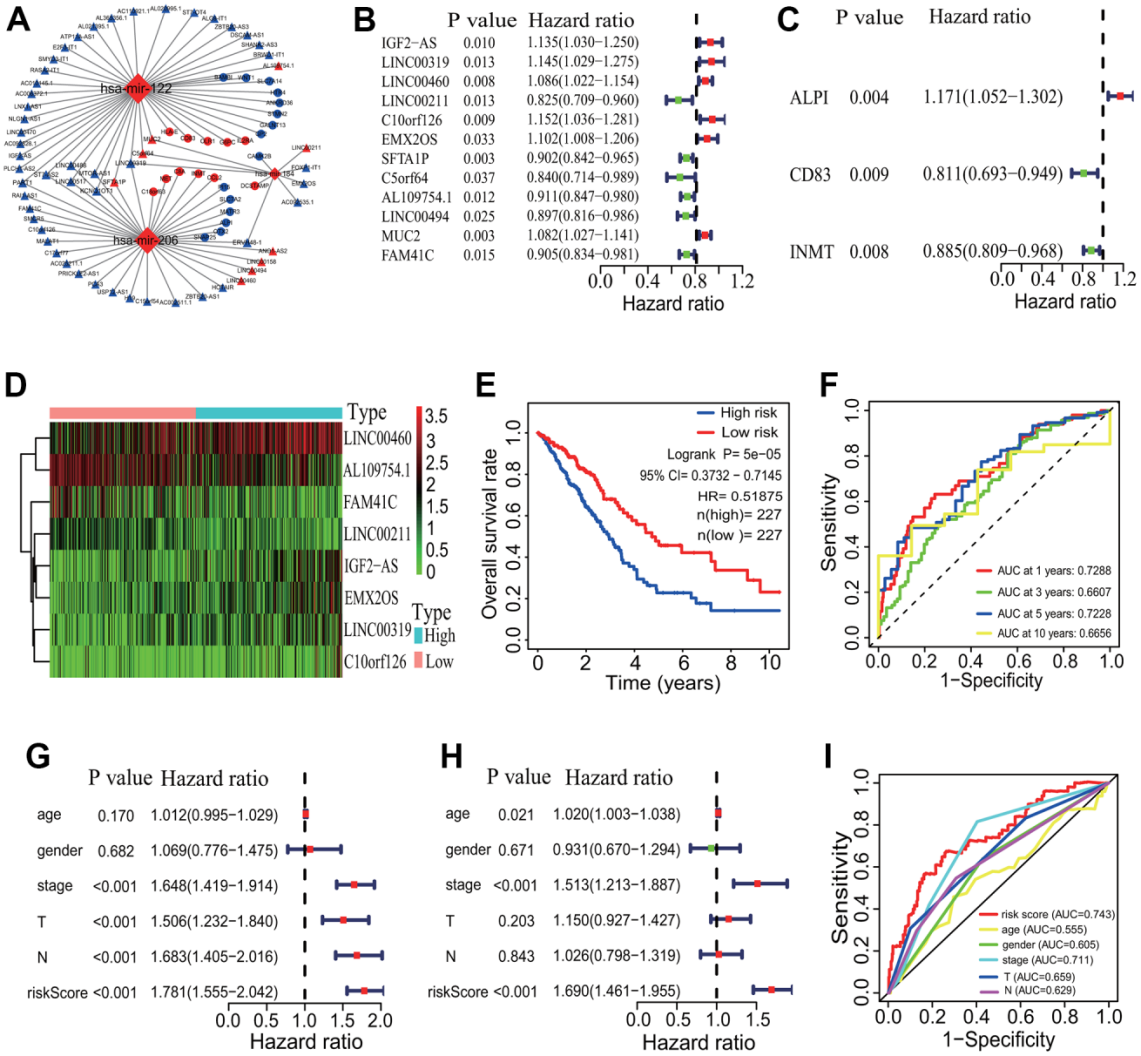
**CeRNA network and prognostic signature construction.** (**A**) CeRNA network. A ceRNA network with 26 DEmRNAs, 3 DEmiRNAs and 57 DElncRNAs was established. (**B**) Univariate Cox regression analysis based on 57 DElncRNAs. Twelve DElncRNAs were identified to have significant associations with the OS of LUAD patients. (**C**) Univariate Cox regression analysis based on 26 DEmRNAs. Three DEmRNAs were identified to have significant associations with the OS of LUAD patients. (**D**) Gene expression profiles of 8 DElncRNAs. Five DElncRNAs and 3 DElncRNAs were separately highly and lowly expressed in the high-risk group. (**E**) Survival curve. The patients in the low-risk group exhibited a better overall survival rate than those in the high-risk group (*p*=5e-05, HR=0.51875, 95% CI=0.3732-0.7145). (**F**) ROC curve correlated with survival. The AUCs of 1-, 3-, 5- and 10-years of the 8-lncRNA prognostic model were separately 0.7288, 0.6607, 0.7228 and 0.6656. (**G**, **H**) Independent prognostic analysis. The 8-lncRNA risk signature was significantly correlated with the OS of LUAD patients by a univariate and a multivariate Cox regression analysis. (**I**) ROC curve correlated with independent prognostic signature. The AUCs of risk score independently predicting survival was 0.743. CeRNA, competitive endogenous RNA; DElncRNA, differentially expressed lncRNA; DEmRNA, differentially expressed mRNA; DEmiRNA, differentially expressed miRNA; LUAD, lung adenocarcinoma; ROC, receiver operating characteristic; AUC, area under the curve; OS, overall survival.

To investigate the interactive pattern among differentially expressed ceRNAs, a ceRNA network was established according to the ceRNA hypothesis. The interactive relationships among DElncRNAs, DEmiRNAs and DEmRNAs were predicted using the online miRcode, miRDB, TargetScan and miRTarBase tools. Finally, totals of 26 DEmRNAs (11 upregulated and 15 downregulated), 3 DEmiRNAs (all upregulated) and 57 DElncRNAs (9 upregulated and 48 downregulated) were filtered into the ceRNA network (Figure 8A). In the ceRNA regulatory network, three upregulated DEmiRNAs were separately *mir-122*, *mir-206* and *mir-184*. Among, *mir-122* and *mir-206* had the most of target DEmRNAs and DElncRNAs (separately 46 and 39 nodes). Among the 26 DEmRNAs, 5 upregulated genes including *IL2RA*, *CCL2*, *DCSTAMP*, *CD83* and *HLA-E* were immune-related genes.

The survival analysis showed that 11 of the 57 DElncRNAs (*AC110921.1*, *ATP11A-AS1*, *C10orf126*, *H19*, *HOTAIR*, *LINC00460*, *LINC00488*, *MALAT1*, *MUC2*, *NLGN1-AS1* and *SFTA1P*) and 3 of the 26 DEmRNAs (*CD83*, *GALNT13* and *PI15*) were significantly associated with the OS of LUAD patients (Supplementary Figure 2). No DEmiRNAs were found the significant association with the survival. A univariate regression analysis showed that 12 of the 57 DElncRNAs (*IGF2-AS*, *LINC00319*, *LINC00460*, *LINC00211*, *C10orf126*, *EMX2OS*, *SFTA1P*, *C5orf64*, *AL109754.1*, *LINC00494*, *MUC2* and *FAM41C*) and 3 of the 26 DEmRNAs (*ALPI*, *CD83* and *INMT*) had significantly prognostic values (Figure 8B, 8C). A multivariate Cox regression analysis showed that a group of 8 DElncRNAs (*IGF2-AS*, *LINC00319*, *LINC00460*, *LINC00211*, *C10orf126*, *EMX2OS*, *AL109754.1* and *FAM41C*) had significantly prognostic value for the survival of LUAD patients. An 8-lncRNA prognostic signature model was established, and 5 DElncRNAs (*IGF2-AS*, *LINC00319*, *LINC00460*, *C10orf126* and *EMX2OS*) and 3 DElncRNAs (*LINC00211*, *AL109754.1* and *FAM41C*) were separately highly and lowly expressed in the high-risk group (Figure 8D). The mortality rate of LUAD patients was significantly lower in the low-risk group (*p*=5e-05, HR=0.51875, 95% CI=0.3732-0.7145, Figure 8E). The AUCs of 1-, 3-, 5- and 10-year correlated with the survival of the 8-lncRNA signature were separately 0.7288, 0.6607, 0.7228 and 0.6656 (Figure 8F). No DEmRNA signature was found to have significantly prognostic value for the OS of LUAD patients.

The independently prognostic power of the 8-lncRNA signature was evaluated. A univariate regression analysis showed that the risk score and some clinical features including pathological stage, T stage and M stage were significantly correlated with the survival of LUAD patients (all *p* <0.001, Figure 8G). A multivariate Cox regression analysis showed that age (*p*=0.021), pathological stage and risk score (both *p* <0.001) were significantly related to the survival of LUAD patients (Figure 8H). The AUCs of the risk score and clinical features were showed in Figure 8I, which showed a higher statistical power of risk score and pathological stage in predicting the survival of LUAD patients (risk score AUC=0.743, pathological stage AUC=0.711).

### Key gene coexpression module and gene identification in LUADs with differing immune phenotypes

**Figure 9:**
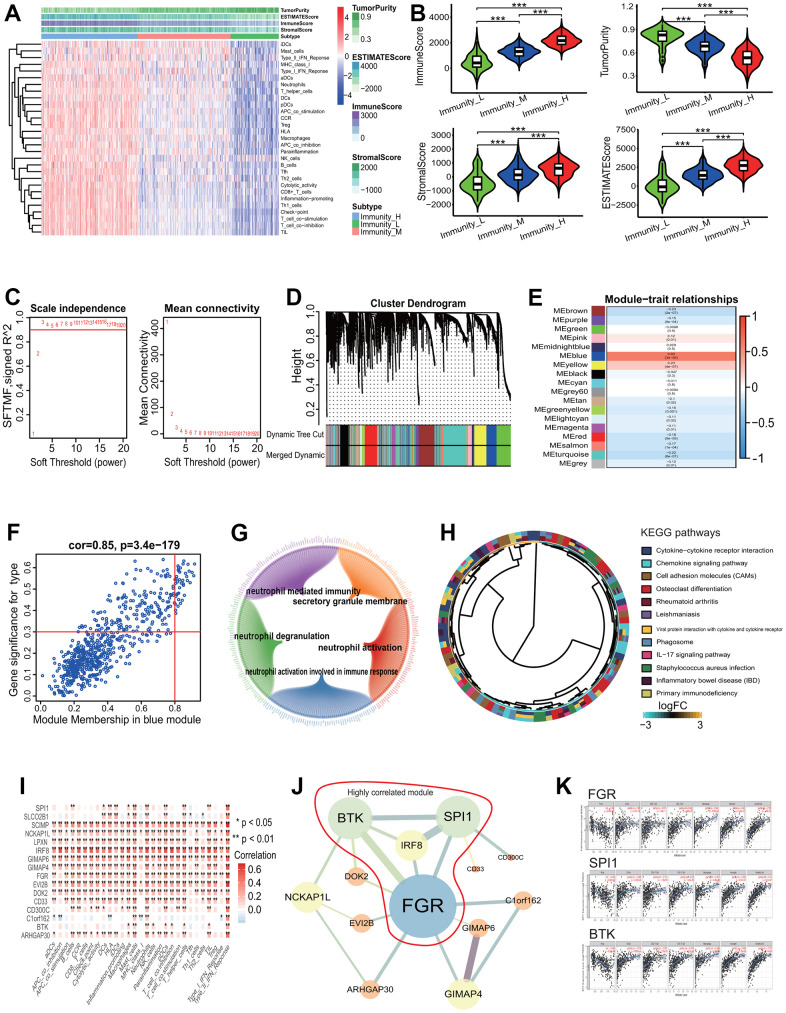
**Gene coexpression analysis based on LUAD with differing immune phenotype.** (**A**) Unsupervised clustering of LUAD patients from the TCGA cohort using ssGSEA scores from immune cell types. The group with higher immune infiltration had a higher immune score. (**B**) ssGSEA scores in differing TME immune phenotypes. The group with higher immune infiltration had higher immune score, stromal score and ESTIMATE score and lower tumor purity. (**C**) Analysis of network topology for various soft-threshold powers. The power parameter β=3 was selected to strengthen the correlation adjacency matrix on the basis of a scale-free topology criterion R^2^>0.9. (**D**) Identification of gene coexpression modules associated with immune phenotype. Eighteen gene coexpression modules were identified according to the TOM-based dissimilarity measure. (**E**) Associations of identified modules and immune phenotype. The blue module was the most significant association with the immune phenotype. (**F**) Correlations of module memberships and gene significance in blue module. The Pearson correlation coefficient was 0.85 and the *p* value was 3.4e-179. (**G**) Relationship network of the most significant module genes and top 5 GO enrichment terms. (**H**) The KEGG pathways enriched by genes in the most significant module. (**I**) Correlations of immune-related terms and 16 genes associated with survival. There were very strong positive correlations between them. (**J**) Interaction relationship network of 13 genes among 16 genes associated with survival. Bigger nodes represented more links. Thicker edges represented more combined score. The genes within the red line were highly correlated module genes in the whole network. (**K**) Correlations of 3 key genes and immune infiltration of some types of cells. The expressions of 3 genes were significant positively correlated with the immune infiltration levels of some types of cells. LUAD, lung adenocarcinoma; TCGA, the cancer genome atlas; ssGSEA, single-sample gene set enrichment analysis; KEGG, Kyoto Encyclopedia of Genes and Genomes; GO, gene ontology, TOM, topological overlap measure.

To elucidate the gene coexpression characteristic associated with the immune phenotype in LUAD and identify key genes, a WGCNA was performed. The LUAD patients were first divided into 3 immune infiltration subgroups (high immune infiltration: 201; intermediate immune infiltration: 195; and low immune infiltration: 101) according to the immune infiltration (Figure 9A). The tumor purity score, immune score, stromal score and ESTIMATE score showed a better grouping (Kruskal-Wallis test, all *p*<0.001, Figure 9B). In the light of the scale-free topology criterion R^2^>0.9, the power β=3 was selected to construct a strengthened adjacency matrix (Figure 9C). In accordance with the calculated TOM-based dissimilarity, 18 gene coexpression modules were identified (Figure 9D). Among these modules, the blue module was the most significant module correlated with the immune phenotype (cor=0.63, *p*=3e-55, Figure 9E), and the correlation between the gene significance (GS) and the module membership (MM) in the blue module was the highest across all modules (cor=0.85, *p*=3.4e-179, Figure 9F). The blue module included 638 genes (Supplementary Table 5) and these genes were significantly enriched in 101 GO terms according to a adjust *p*<0.0001 (including 93 BPs, 7 CCs and 1 MF). The first 5 GO terms were separately neutrophil activation (BP, adjust *p*=1.08E-13), neutrophil mediated immunity (BP, adjust *p*=1.08E-13), neutrophil activation involved in immune response (BP, adjust *p*=1.18E-13), secretory granule membrane (CC, adjust *p*=1.84E-13) and neutrophil degranulation (BP, adjust *p*=2.98E-13). The relationships between genes and GO terms were showed in Figure 9G. These genes were mainly enriched in 12 KEGG pathways including some immune pathways, such as chemokine and IL-17 signaling pathways (Figure 9H). According to the MM score>0.8, 39 genes were selected as hub genes and used to explore the relationships between these genes and the OS of LUAD patients. The results showed that 16 genes (*BTK*, *SCIMP*, *GIMAP4*, *CD300C*, *CD33*, *LPXN*, *GIMAP6*, *IRF8*, *DOK2*, *ARHGAP30*, *C1orf162*, *SLCO2B1*, *EVI2B*, *FGR*, *NCKAP1L*, *SPI1*) had significant associations with the OS of LUAD patients (all *p*< 0.05, Supplementary Figure 3). The expressions of 16 genes had very strong positive correlations with the immune scores of 29 immune-related terms (Figure 9I). The interaction relationship analysis showed that 13 of the 16 genes (*BTK*, *GIMAP4*, *CD300C*, *CD33*, *GIMAP6*, *IRF8*, *DOK2*, *ARHGAP30*, *C1orf162*, *EVI2B*, *FGR*, *NCKAP1L*, *SPI1*) had 20 gene-gene interaction pairs and a PPI network with 13 genes and 20 edges was established (Figure 9J). A highly correlated module containing 4 genes (*FGR*, *BTK*, *SPI1*, *IRF8*) was identified from the whole PPI network (Figure 9J). Among the 4 genes, the expressions of 3 genes (*FGR*, *BTK*, *SPI1*) had significant positive correlations with the immune infiltration of some types of immune cells such as dendritic cell (*FGR*: cor=0.708 and *p*=1.78e-75, *SPI1*: cor=0.744 and *p*=5.01e-87, *BTK*: cor=0.752 and *p*=3.74e-90, Figure 9K).

### Evaluation of predictive performance of prognostic signature

**Figure 10:**
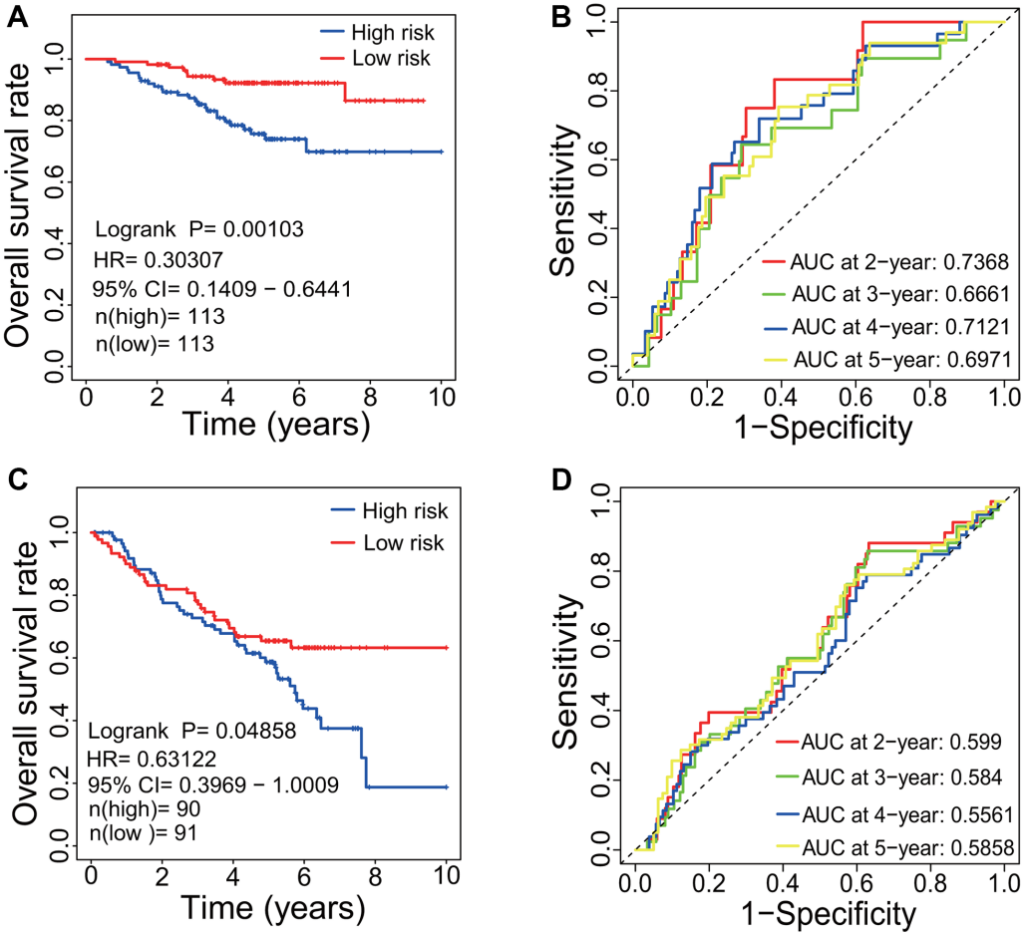
**Evaluation of prognostic signature.** (**A**) Survival curve based on GSE31210 dataset. LUAD patients in the low-risk group had a higher OS rate than that in the high-risk group (*p*=0.00103, HR=0.30307, 95% CI=0.1409-0.6441). (**B**) ROC curve based on GSE31210 dataset. The AUCs of 2-, 3-, 4- and 5-year associated with the survival were separately 0.7386, 0.6661, 0.7121 and 0.6971. (**C**) Survival curve based on GSE50081 dataset. LUAD patients in the low-risk group had a higher OS rate than that in the high-risk group (*p*=0.04858, HR=0.63122, 95% CI=0.3969-1.0009). (**D**) ROC curve based on GSE50081 dataset. The AUCs of 2-, 3-, 4- and 5-year associated with the survival were separately 0.599, 0.584, 0.5561 and 0.5858. LUAD, lung adenocarcinoma; OS, overall survival; HR, hazard rate; ROC, receiver operating characteristic; AUC, area under the curve; CI, confidence interval.

To evaluate the predictive performance of the identified prognostic signatures, the LUAD-related microarray dataset GSE31210 and the 8-mRNA prognostic signature (*KCNJ18*, *RPE65*, *GRIA1*, *LCN15*, *C11orf21*, *ANXA13*, *FSIP2* and *KRT76*) were employed. Since the *KCNJ18* gene was not included in the GSE31210 gene expression profiles, it was removed from the 8-mRNA signature during evaluation. The result showed that the LUAD patients had a lower HR and a higher OS rate in the low risk group (HR=0.30307, 95% CI=0.1409-0.6441, *p*=0.00103, Figure 10A). The AUCs of 2-, 3-, 4- and 5-year correlated with the survival were separately 0.7368, 0.6661, 0.7121 and 0.6971 (Figure 10B), which demonstrated the effectiveness as a prognosticator in predicting the OS of LUAD patients.

Through WGCNA, three genes including *FGR*, *BTK* and *SPI1* were identified to have associations with the OS and with the immune infiltration level in LUAD patients. The predictive performance of three genes were further evaluated using the gene expression dataset GSE50081. The result showed that the LUAD patients had a lower HR and a higher OS rate in the low risk group (HR=0.63122, 95% CI=0.3969-1.0009, *p*=0.04858, Figure 10C). The AUCs of 2-, 3-, 4- and 5-year correlated with the survival were separately 0.599, 0.584, 0.5561 and 0.5858 (Figure 10D).

## DISCUSSION

Accumulated evidence has shown the important roles of TME in modulating the cancer progression, guiding the therapy and predicting the prognosis of patients with LUAD. It is crucial to reveal the transcriptome characteristics in LUADs with differing TME immune phenotypes for better understanding the role of TME in LUAD biology. In the current study, we depicted the immune landscape of LUAD, analyzed the gene mutation profile using a large cohort, mined the key DEGs related to the LUAD immunophenotype, constructed the PPI and immune PPI networks, established the ceRNA regulatory network, identified essential genes associated with the LUAD immunophenotype, and predicted some prognostic signatures. Finally, we systematically revealed the transcriptome characteristics in LUADs with differing TME immune phenotypes and identified some key genes and built three robust prognostic signatures including a 9-lncRNA, an 8-lncRNA and an 8-mRNA.

Among identified key genes, five genes including *FPR2*, *KNG1*, *GNGT2*, *ADCY8* and *PPBP* were identified to have association with LUAD immune phenotype by a PPI network based on 1169 key DEGs. FPR2 belongs to the formyl peptide receptor family and is a G-protein coupled receptor. FPR2 has been identified to promote the invasion and metastasis in various tumors including colorectal cancer and gastric cancer [13, 14], and serves as a prognosticator in gastric cancer [14]. *KNG1* is a protein coding gene, and has association with some diseases including angioedema and high molecular weight kininogen deficiency. GNGT2 belongs to the G protein gamma family and is thought to play a vital role in cone phototransduction. A recent study showed that GNGT2 was closely associated with the survival of esophageal cancer, and serve as a potentially prognostic marker of patients with esophageal cancer [15]. ADCY8 is an adenylate cyclase and catalyzes the formation of cyclic AMP from ATP. *PPBP* gene encodes a protein of platelet-derived growth factor, and plays the key roles in various cellular processes including glycolysis, mitosis and DNA synthesis. Five key immune genes including *IL2RA*, *CCL2*, *DCSTAMP*, *CD83* and *HLA-E* were identified by constructing a ceRNA network. Some published results showed that *IL2RA* was abnormally expressed in a few types of cancers including head and neck, leukemia, breast, lymphoma, lung and prostate [16]. The high expression of *IL2RA* results in a lower survival rate for the patients [16]. *CCL2* is a cytokine gene and play the roles in the immunoregulatory and inflammatory processes. A study showed that the epigenetic silencing of *CCL2* potentiates tumor development by repressing the macrophage infiltration in small cell lung cancer [17]. Another study showed that the immunotherapeutic effect of anti-PD1 was enhanced by blocking the expression of *CCL2* in LC [18]. DCSTAMP is a seven-pass transmembrane protein expressed by dendritic cells and associates with some diseases such as Paget's disease of bone [19, 20]. CD83 is the dendritic cell maturation marker and have immunosuppressive properties [21]. The CD83 expression in cancer cells facilitates the tumor growth [21]. *HLA-E* is a major histocompatibility complex gene, and is expressed in many types of cancers and served as a potential prognosticator of some cancers such as melanoma and colorectal cancer [22, 23]. A group of 8 immune genes (*IL17A*, *FOXP3*, *CTLA4*, *TLR4*, *IFNG*, *CCL2*, *CD19* and *CXCL9*) were identified as key immune genes associated with LUAD immune phenotype by constructing an immune PPI network. Presently, some studies have showed that some genes had the associations with several types of cancers. For example, the high expression of *CTLA4* showed a poor survival and serves as an independent risk factor to evaluate the prognosis of breast cancer [24]. *TLR4* promotes the immune escape of NSCLC by upregulating *PD-L1* [25]. *CXCL9* promotes the progression of prostate cancer by inhibiting the cytokines from T cells [26]. In summary, some key genes were identified by a variety of bioinformatics methods in this study and the roles of a few genes have been deeply studied in tumor biology, such as *FPR2* and *TLR4*. Nevertheless, we have little understanding of the roles of some genes such as *KNG1* and *DCSTAMP*. Next, the roles of these genes in tumor biology should be focused, especially clarified that how the genes play the roles through the immune pathways.

In the identified three robust prognostic signatures, the 9-lncRNA (*AP003555.2*, *LINC02310*, *AC026462.3*, *AL162293.1*, *AC078860.1*, *AC034223.1*, *HSPC324*, *AC105999.2* and *AP005137.2*) signature was identified to significantly associate with the OS of LUAD patients based on 1085 key DElncRNAs. The lncRNA *HSPC324* has been identified to play a regulatory role in lung development and tumorigenesis [27]. Other eight lncRNAs were first identified to have the associations with the OS of LUAD patients. Furthermore, the 9-lncRNA signature was identified to serve as an independent prognostic marker to predict the survival of LUAD patients, which provides some new insights for evaluating the clinical outcomes of LUAD patients. The 8-lncRNA (*IGF2-AS*, *LINC00319*, *LINC00460*, *LINC00211*, *C10orf126*, *EMX2OS*, *AL109754.1* and *FAM41C*) prognostic signature was identified on the basis of the ceRNA network analysis. Some published results showed that some lncRNAs play an important regulatory role in the tumorigenesis of some cancers such as breast cancer, ovarian cancer, cervical cancer and so on. For example, the lncRNA *IGF2-AS*, as an antisense gene of *IGF2*, inhibits the tumorigenesis of breast cancer by epigenetically regulating *IGF2* and affects the metastasis and prognosis of gastric adenocarcinoma by sponging miR-503 to regulate *SHOX2* [28, 29]. The lncRNA *EMX2OS* has been reported to affect the proliferation and invasion of ovarian cancer cells by sponging miR-654-3p to regulate *AKT3* and the downregulation of *EMX2OS* results in a poor prognosis of patients with kidney renal clear cell carcinoma [30, 31]. The lncRNA LINC00319 separately promotes the progression of cervical cancer via regulating the miR-147a/*IGF1R* axis and the osteosarcoma progression via sponging miR-455-3p to regulate *NFIB* [32, 33]. The lncRNA *FAM41C* has been shown to have the association with the recurrence of papillary thyroid cancer [34]. Furthermore, two lncRNAs *LINC00319* and *LINC00460* have been reported to have associations with lung cancer [35, 36]. Despite this, few published studies showed the regulatory roles of these lncRNAs in LUAD. Our results showed that these lncRNAs were significantly dysregulated in LUAD tissue and had significant associations with the survival of patients with LUAD. In the 8-mRNA signature (*KCNJ18*, *RPE65*, *GRIA1*, *LCN15*, *C11orf21*, *ANXA13*, *FSIP2* and *KRT76*), the expressions of some mRNAs have been reported to have significant associations with the survival in some cancers. For example, *GRIA1* serves as a prognosticators for basal-like bladder cancer [37]. *KRT76* was downregulated expressed in human oral squamous cell carcinomas and correlated with a poor prognosis [38]. From the expression profiles, we predicted the prognosis of LUAD patients based on the 8-mRNA signature.

In the established immune prognostic signature based on the 88 immune DEGs, three immune genes including *HLA-DRB5*, *CX3CR1* and *INHA* were found to significantly associate with the OS of LUAD patients. *HLA-DRB5* is a major histocompatibility complex gene. *CX3CR1* gene encodes a receptor for the chemokine fractalkine. *INHA* gene encodes a protein belonging to the transforming growth factor-beta (*TGF-beta*) superfamily. Three genes have been mainly implicated in regulating immune response. A few not many studies have shown the associations of the three genes with tumors [39, 40].

Four key genes including *FGR*, *BTK*, *SPI1* and *IRF8* were identified using WGCNA method. Among, *FGR* and *BTK* are tyrosine kinase genes and *SPI1* and *IRF8* are transcription factor genes. *FGR* encodes a *Src* family tyrosine kinase and has been reported to have association with the tumor progression as a proto-oncogene in some cancers such as colorectal cancer [41]. In addition, a few studies have also showed the associations between *FGR* and lung diseases [42]. However, few published reported that *FGR* had an association with lung cancer. The current results identified that *FGR* plays a potential role in LUAD and was as a prognostic factor. *BTK* is a gene encoding bruton tyrosine kinase and plays a vital role during the B-cell development. Some studies showed that *BTK* played a potential role in LUAD and was as a potential prognostic factor and an indicator for TME remodeling in LUAD [43]. *SPI1*, an ETS-domain transcription factor, plays the roles by activating the gene expression of target genes during the myeloid and B-lymphoid cell development. A recent study demonstrated that *SPI1* promoted NSCLC by a ceRNA regulatory mechanism [44]. *IRF8*, a transcription factor gene, belongs to the interferon regulatory factor family. A study has shown that *IRF8* inhibits the tumor by inducing the senescence of lung cancer cells [45]. *IRF8* was aberrantly expressed by a higher methylation level in NSCLC tissues compared with non-malignant lung tissues [46]. Despite some valuable researches, there is still a lack of available information about the roles of the 4 genes in LUAD. Our results demonstrated that two kinase genes and two transcription factor genes played the key roles and were as predictors to predict the prognosis of LUAD patients.

To summarize, although the transcriptome characteristics of LUADs with differing immune phenotypes were systematically analyzed and some potentially prognostic signatures were identified, some limitations must be noted. First, some identified key genes are obtained by bioinformatics methods and the expressions of some genes have been validated by analyzing 20 transcriptome sequencing data. Nevertheless, these genes must be verified using some more accurate quantitative methods such as qPCR. Second, although some prognostic signatures were identified by various methods and the predictive performances of two signatures were well evaluated using two independent datasets, the robustness of prognostic signatures need be verified using large-scale follow-up data. Last, the clinical application of each prognostic signature must be considered. In theory, the signature with the lowest *p* value in the survival curve and with the highest AUC value in the ROC curve should be first selected to use. If two values are inconsistent, the signature with higher AUC value should be considered because the AUC value represents the reliability of the prognostic model. As a result, the 9-lncRNA prognostic signature (*AP003555.2*, *LINC02310*, *AC026462.3*, *AL162293.1*, *AC078860.1*, *AC034223.1*, *HSPC324*, *AC105999.2* and *AP005137.2*) should be fist selected to use in clinic.

## CONCLUSIONS

The current study systematically revealed the transcriptome characteristics in LUADs with differing immunity by a comprehensive bioinformatics method and identified some key genes related to differing TME immune phenotype and three robust prognostic signatures associated with the survival of LUAD patients. The findings provide novel insights into the immunological mechanism in LUAD biology and in predicting the prognosis of LUAD patients.

## MATERIALS AND METHODS

### Gene expression data related to LUAD and clinical information collection

LUAD related gene expression profiles were retrieved from The Cancer Genome Atlas (TCGA) database (2020, https://portal.gdc.cancer.gov/), and the inclusion criteria of gene expression profiles were as follows: (1) histological diagnosis for LUAD; (2) no other malignancy or malignancies besides LUAD; (3) complete clinical data. Finally, the gene expression profiles including 497 LUAD tissues and 54 non-LUAD normal lung tissues were collected in the current study. Furthermore, the clinical data of the 497 LUAD patients were retrieved from the TCGA database.

The miRNA dataset including 483 LUAD and 45 non-LUAD normal lung tissues were retrieved from the TCGA database. The miRNA data were used to elucidate the regulatory relationships among lncRNAs, miRNAs and mRNAs by constructing a ceRNA network on the basis of the ceRNA hypothesis.

GSE31210 and GSE50081 datasets were retrieved from the GEO database (https://www.ncbi.nlm.nih.gov/geo/), and were used to evaluate the predictive performance of two identified prognostic signatures.

All data used in this study have been approved by the Institutional Review Board of the relevant participating institutions, and no additional approval was required from the ethics committee. The current study meets the requirements of using and publishing the public data from the TCGA and GEO databases.

### Single-sample gene set enrichment analysis

The single-sample gene set enrichment analysis (ssGSEA) in the R gsva package was used to quantify the infiltration levels of 29 immune cell types [47]. The 29 immune cell types were obtained from the publication of Bindea et al. and Barbie et al. [48, 49], and included Cytolytic_activity, T cell co-inhibition, aDCs, B cells, APC co-inhibition, APC co-stimulation, CCR, Check-point, DCs, T helper cells, HLA, iDCs, Inflammation-promoting, Macrophages, Mast_cells, Neutrophils, CD8+ T cells, NK cells, pDCs, T cell co-stimulation, Tfh, Th1 cells, Th2 cells, MHC class I, TIL, Treg, Type I IFN Response, Parainflammation and Type II IFN Response. The relative abundances of each of the 29 immune cell types were represented according to the enrichment scores from the ssGSEA method between differing immune infiltration groups.

### Evaluation of immune cell types

CIBERSORT (https://cibersort.stanford.edu/), an analytical tool for characterizing cell composition in a complex tissue according to their gene expression profiles, was used to estimate the immune cell composition of LUAD tissues with mixed cell types [50]. The 22 immune cell types were distinguished using a gene signature matrix termed LM22 of 547 genes. The LM22 signature and 1000 permutations were performed by a deconvolution algorithm. The proportions of the immune cell subsets between the high and low immune infiltration subgroups were compared using the Mann-Whitney U test. The significant criterion of the accuracy for distinguishing cell types was set as the CIBERSORT *p*<0.05.

### Data normalization and differentially expressed analysis

The normalization of all raw RNA-seq data were implemented using the trimmed mean of M-values (TMM) method based on the edgeR package (version 3.28.0) in Bioconductor project (http://www.bioconductor.org/, version 3.10) [51]. The edgeR package was further used to screen three types of differentially expressed RNAs (DElncRNA, DEmRNA, DEmiRNA) between the LUAD tissues and non-LUAD lung tissues as well as between differing immune infiltration subgroups. A |Log fold change (logFC)| >1 and an adjusted *p* value <0.05 (*p*<0.05) were set as the significant criteria of gene differential expression.

### PPI network construction and key gene identification

PPI network was used to elucidate the interactive relationships among DEGs encoding proteins and was constructed on the basis of the interactive relationships between all gene pairs from the online STRING database (https://string-db.org/, version 11.0) [52]. A gene pair with a combined score ≥0.9 indicates a strong interaction and was filtered into the PPI network. The cytoscape software (http://www.cytoscape.org/, version 3.7.2) was used to construct the PPI network [53]. The highly correlated module was the subnetwork with a stronger interactive relationship among genes in the whole PPI network, and was extracted from the PPI network using the molecular complex detection (MCODE) algorithm on the basis of the topological properties of the whole PPI network. The MCODE analysis was implemented using a plugin MCODE (version 1.5.1) in the cytoscape software [54]. The Node Score Cutoff=0.4, Max. Depth=100, K-Core=4 and Degree Cutoff=4 were set as the threshold parameters. The module with the highest score was the most critical module, and was used to identify key genes using seven centrality methods based on a plugin CytoNCA (version 2.1.6) in the cytoscape software [55]. The genes with higher centrality scores obtained from each centrality method were identified as key genes, and the intersecting genes of key genes obtained from seven centrality methods were identified as essential genes associated with differing immune phenotypes.

### CeRNA network construction

The lncRNAs-miRNAs-mRNAs interactive relationships were elucidated by constructing a lncRNA-miRNA-mRNA regulatory network on the basis of the ceRNA hypothesis [56]. The ceRNA network was established as follows: (1) keeping three types of key DERNAs with the |logFC| >1 and *p*<0.05 and the key DERNAs were intersecting DERNAs between the high and low immune infiltration groups and between the LUAD and normal lung tissue groups; (2) predicting the potential interactive relationships between key miRNAs and lncRNAs using the online miRcode tool (miRcode 11) [57]; (3) predicting the potential interactive relationships between key mRNAs and miRNAs using three online tools including the TargetScan (http://www.targetscan.org/, release 7.2) [58], the miRDB (http://mirdb.org/, version 6.0) [59] and the miRTarBase (release 7.0) [60]; (4) on the basis of the ceRNA hypothesis, the intersecting miRNAs between the potential lncRNA-miRNA pairs and miRNA-mRNA pairs were chosen to construct the ceRNA network. The cytoscape software (version 3.7.2) was used to build the lncRNA-miRNA-mRNA ceRNA network [53].

### Survival analysis and prognostic model construction

Survival analysis was implemented using a Kaplan-Meier (KM) estimate method in the survival package (version 2.43-3), and the log-rank (LR) *p* value and the hazard ratio (HR) with 95% confidence interval (CI) were computed. Subsequently, the associations between DERNAs and the OS of LUAD patients were evaluated by a univariate Cox proportional hazards regression model. Next, a prognostic model was constructed in line accordance with a multivariate Cox hazards regression model. The risk score formula was established as follows:


$$\begin{array}{l} \text{Risk}\;\text{score}=\sum {}_{i}Expression\;(DERNA_{i})\\ \;\;\;\;\;\;\times Coefficient\;(DERNA_{i}) \end{array}$$

where “*Expression*(*DERNA_i_*)” represents the expression of the *i*th DERNA, and “*Coefficient*(*DERNA_i_*)” denotes the regression coefficient of the *i*th DERNA from a multivariate Cox regression model. In the light of the median risk sore, the patients were separated into the high- and low-risk groups. The receiver operating characteristic (ROC) curve was used to measure the risk prediction rate of risk signature and the ROC curve was constructed by the survivalROC (version 1.0.3) package. The area under curve (AUC) in the ROC curve shows the prediction accuracy of risk signature.

### WGCNA

The coexpression relationships among genes associated with immune phenotype were analyzed using the WGCNA method, and gene coexpression modules were identified using the WGCNA package (version 1.13) [61]. First, the Pearson correlation coefficients for all pair-wise genes were computed according to the expressions of all genes and a Pearson correlation matrix was constructed based on the Pearson correlation coefficients. Subsequently, an adjacency matrix was converted by the Pearson correlation matrix and the adjacency matrix was strengthen using a power adjacency parameter β (soft threshold). The power β=3 was selected on the basis of the scale-free topology criterion R^2^=0.9. Next, a topological matrix was computed from the strengthened adjacency matrix using the topological overlap measure (TOM) that was defined as the correlation between each pair of genes. Based on the TOM-based dissimilarity (1-TOM), the genes with coherent expression profiles were classified into one gene module by an average linkage hierarchical clustering. A dynamic cutting algorithm was used to construct a system cluster tree of all genes, and gene coexpression modules associated with immune phenotype were identified from the system cluster tree. The gene coexpression modules with 95% similarity were integrated into one module. The first principal component was the representative of gene expression in a module, defining as the module eigengene (ME). The correlation between the gene module and the ME was calculated and was defined as the module membership (MM). The gene significance (GS) indicated the correlation between the gene expression and immune phenotype, and was calculated using the log10 transformation of the *p* value from t-test measuring gene differential expression. The average GS of all genes in one module showed the correlation between the module and immune phenotype, and was defined as the module significance (MS).

## Supplementary Material

Supplementary Figures

Supplementary Table 1

Supplementary Tables 2 and 3

Supplementary Table 4

Supplementary Table 5
