# Supplementary material for: Identification of prognostic candidate signatures by systematically revealing transcriptome characteristics in lung adenocarcinoma with differing tumor microenvironment immune phenotypes
Source: Aging (Albany NY). 2022 Jun 7;14(11):4786–818. doi: 10.18632/aging.204112 (PMC9217709; doi:10.18632/aging.204112)
Supplement: Supplementary Figures [file aging-14-204112-s001.pdf]

SUPPLEMENTARY FIGURES

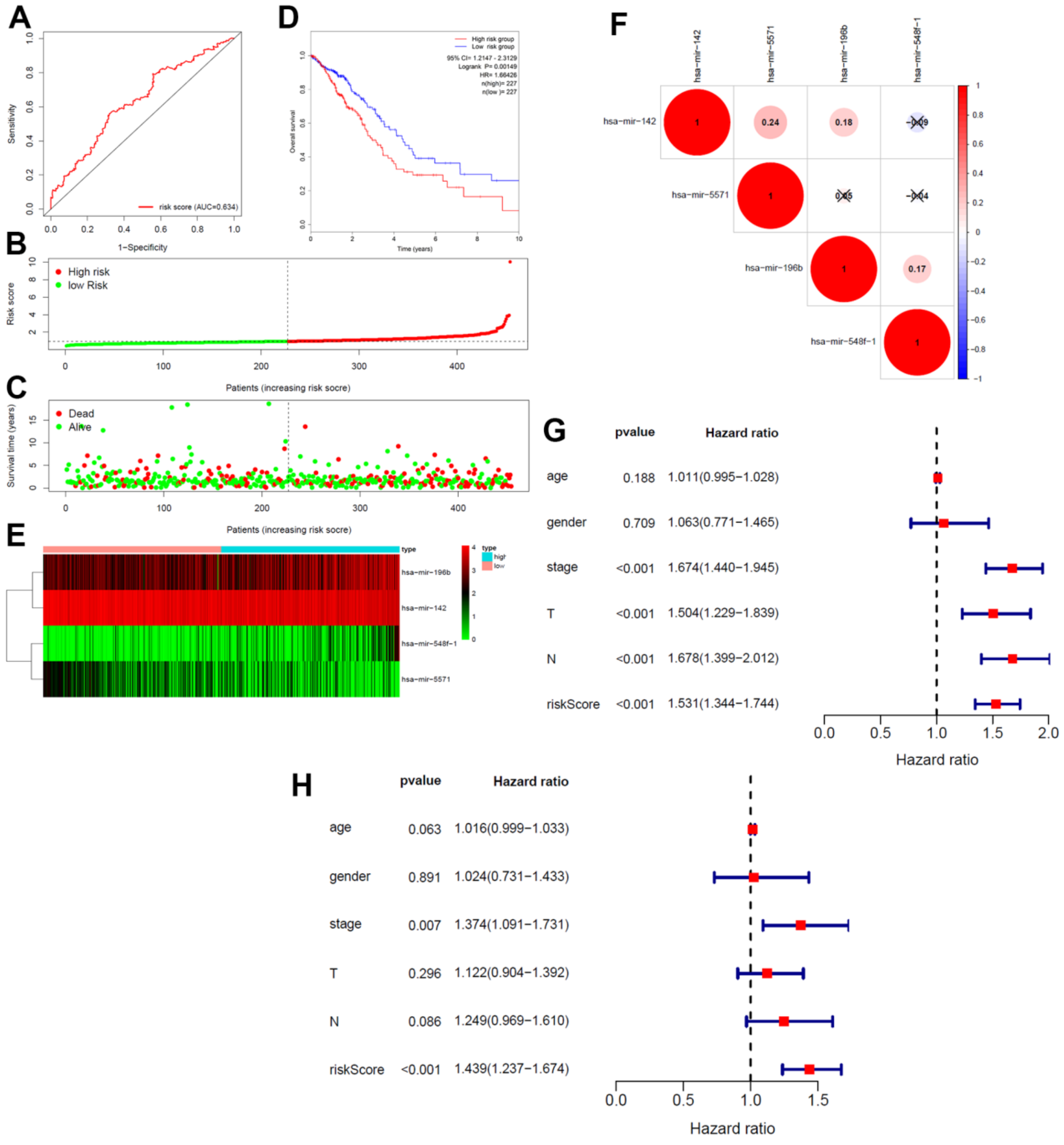

Supplementary Figure 1. Prognostic signature analysis of key DEmiRNAs. (**A**) ROC curve. The 3-year AUC of the 4-miRNA signature (mir-196b, mir-142, mir-548f-1, mir-5571) was 0.634 in the ROC curve. (**B**) Risk score distribution. According to the median risk score, the patients were divided into the high- and low-risk groups. (**C**) Survival overview. The distribution of survival times of LUAD patients in the high- and low-risk subgroups. (**D**) Survival curve. LUAD patients had a higher HR and a lower OS in the high-risk group (p=0.00149, HR=1.6643, 95%CI=1.2147-2.3129). (**E**) The expression heatmap of four miRNAs. Two miRNAs (mir-196b and mir-548f-1) were highly expressed and 2 miRNAs (mir-142 and mir-5571) were lowly expressed in the high-risk group. (**F**) The expression correlations among 4 miRNAs. The correlations among 4 miRNAs were low in expression. (**G**, **H**) Independently prognostic analysis. The 4-miRNA risk signature was significantly correlated with the survival of LUAD patients by a univariate (p<0.001, HR=1.531, 95%CI=1.344-1.744) and a multivariate (p<0.001, HR=1.439, 95%CI=1.237-1.674) Cox regression analyses. ROC, receiver operating characteristic; AUC, area under the curve; LUAD, lung adenocarcinoma; HR, hazard ratio; OS, overall survival; CI, confidence interval.

DElncRNAs associated with the OS of LUAD patients

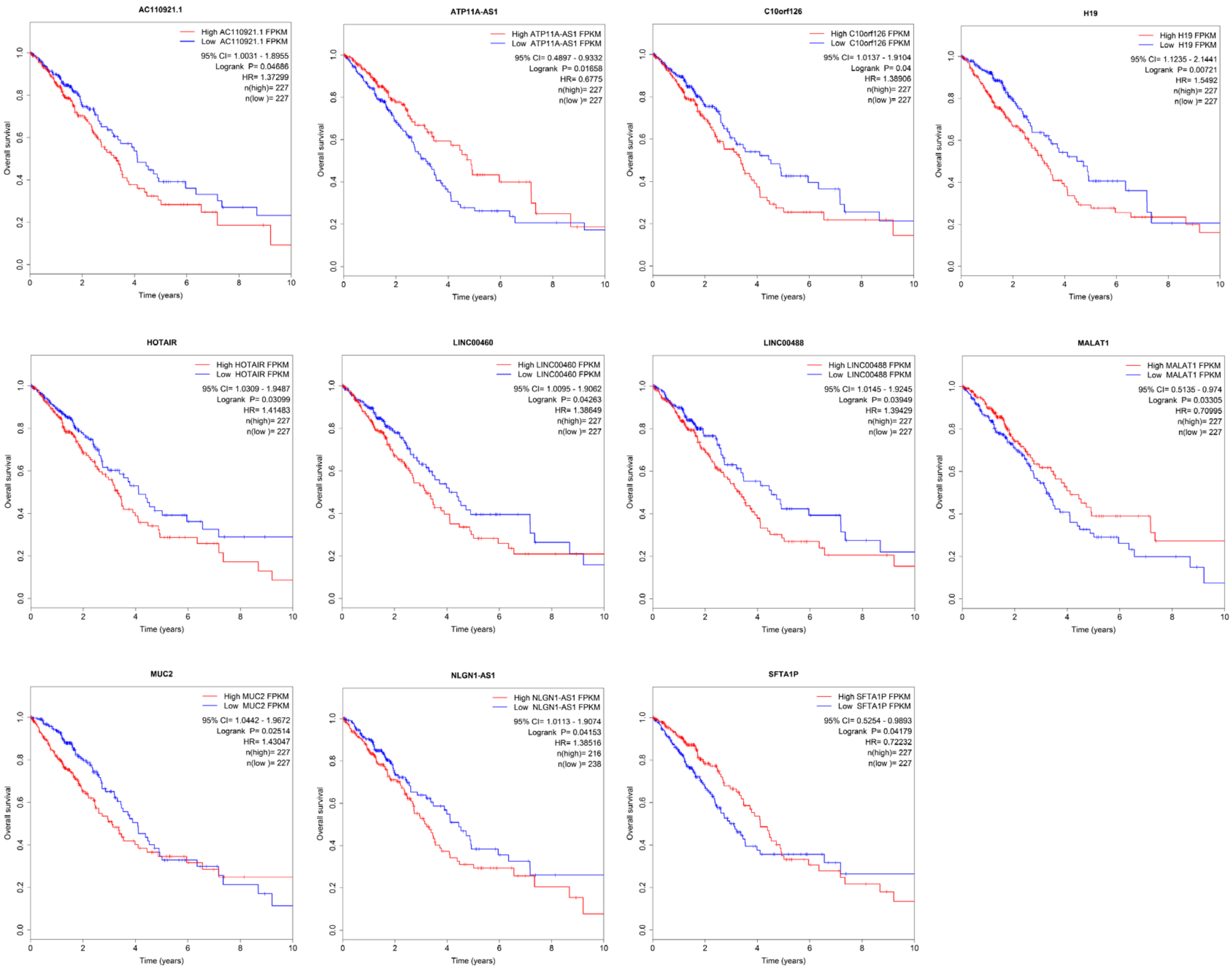

DEmRNAs associated with the OS of LUAD patients

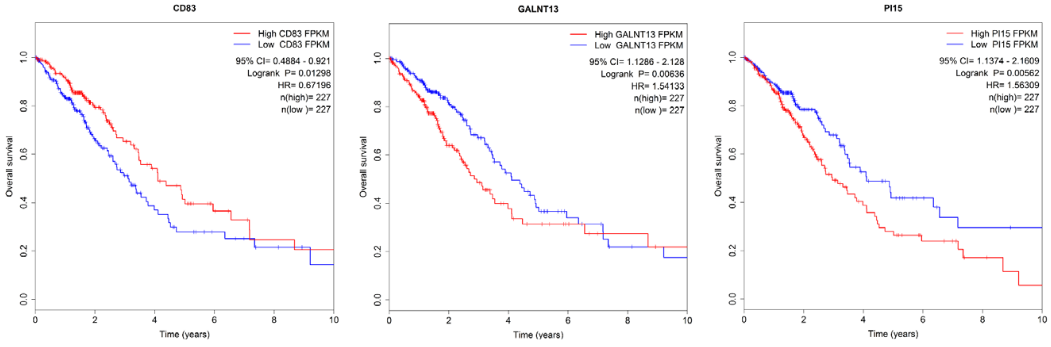

Supplementary Figure 2. Survival curves of DERNAs in the ceRNA network.

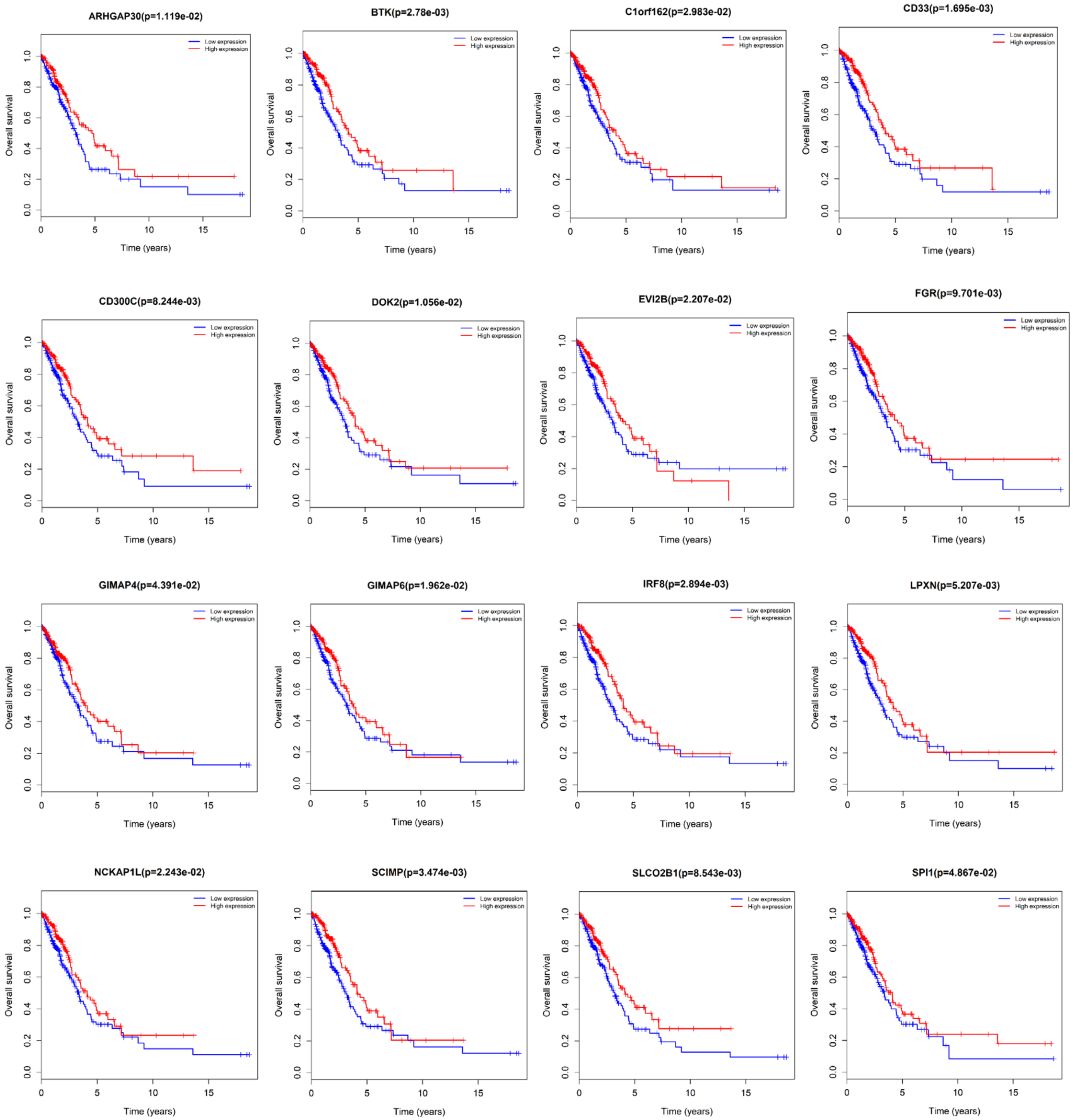

Supplementary Figure 3. Survival curves of 16 genes.
