# Supplementary material for: Identification of prognostic candidate signatures by systematically revealing transcriptome characteristics in lung adenocarcinoma with differing tumor microenvironment immune phenotypes
Source: Aging (Albany NY). 2022 Jun 7;14(11):4786–818. doi: 10.18632/aging.204112 (PMC9217709; doi:10.18632/aging.204112)
Supplement: Supplementary Tables 2 and 3 [file aging-14-204112-s003.pdf]

SUPPLEMENTARY TABLES

Supplementary Table 2. DERNAs associated with the OS of LUAD patients.

lncRNAs				
**id**	**HR**	**HR.95L**	**HR.95H**	**pvalue**
AC022784.1	1.16472348399603	1.08738130821097	1.24756677710763	0.0000136425931594224
PLUT	1.18317343563371	1.09120300736011	1.28289545515089	0.0000462110267742122
AP003555.2	1.17285723888692	1.066619242653	1.28967681043137	0.000997356454309377
LINC02310	1.40733240657435	1.24523155199544	1.59053510924979	4.43375078362903E-08
LINC02448	1.2514134059327	1.10877498385066	1.41240155609337	0.000280950216307133
AL365203.1	1.17789284486517	1.09691948570537	1.26484356606391	6.61663546039384E-06
AC091133.2	1.24680303735831	1.10963673909535	1.40092496868233	0.000207725876635335
AC092168.2	1.29800166283959	1.14631089643021	1.46976559498919	0.0000389762014836297
LINC01468	1.13643666177252	1.06946903893755	1.20759763882803	0.00003669996824663
AC026462.3	1.16507969308909	1.07264844909056	1.26547583450984	0.000291350603204754
AL161668.1	1.40322946480174	1.18566150322529	1.6607209777255	0.0000810873903907402
AL162293.1	1.3668839559206	1.16543159155924	1.60315866026376	0.000122104893615929
AC078860.1	1.26460841206938	1.10637517687766	1.44547208695506	0.000577078162022361
AC034223.1	1.35638496989582	1.20852837753302	1.52233097770931	2.26376942136393E-07
AL078645.1	0.706591952925778	0.592746408803817	0.842303184842592	0.000106840752359197
LINC02178	1.23236024911331	1.15055908083888	1.31997722575648	2.48988246005482E-09
AP000679.1	1.24755964032529	1.101259373755	1.41329562613541	0.000509797325855036
AC105243.1	1.19615135039499	1.09550025769094	1.30604994659473	0.0000650220255123192
HSPC324	0.794536471076285	0.702507838128402	0.898620868846409	0.000250381641626801
AF121898.1	1.21857536234778	1.10736134803875	1.34095877226614	0.0000515487289662321
HOXA10-AS	1.15728628387467	1.06731859745349	1.25483763333638	0.000403493083551291
AC105999.2	1.21966385571151	1.08861395723691	1.36648984797585	0.000617179551695052
AP000924.1	1.18746303654532	1.09086513126322	1.29261484554794	0.0000721841736956064
AP005137.2	1.28183223967341	1.17374333508091	1.39987494842974	3.30984194844202E-08
AC139722.1	1.38094509002328	1.21922351755444	1.56411791127893	3.79354106211565E-07
AC104984.3	0.686835618146608	0.551101904542505	0.855999883989601	0.000825600053680081
**mRNAs**				
**id**	**HR**	**HR.95L**	**HR.95H**	**pvalue**
DLX2	1.16952735712133	1.07060834148525	1.27758600979857	0.000514426041153686
KCNJ18	1.14117877176951	1.06024314881763	1.22829276528659	0.000433923149560757
RHOV	1.19313935255989	1.10102235023076	1.29296332116116	0.0000165076567542321
RPE65	1.20470642228417	1.0909009234457	1.33038439394535	0.000234695890899623
IGF2BP1	1.10242324206862	1.05488816064131	1.15210033631834	0.0000145036964461706
GUCA2B	1.12214601161301	1.04939377223204	1.19994200909033	0.00075250016364406
GRIA1	0.863734356844824	0.801226374681498	0.931118923151657	0.000132374723448509
SFTPC	0.944720442695209	0.913545199496473	0.97695955858359	0.000895462193585035
LCN15	1.13581935440546	1.06228136535799	1.214448118844	0.000192155904015075
C11orf21	0.795087689608751	0.705146616040704	0.896500698984976	0.000181290007184054
ANXA13	1.12179649556213	1.05283943067546	1.19526999159608	0.00038414805431614
FSIP2	1.15393167148808	1.06920874406336	1.24536795069867	0.000233289016522983

KRT76	1.2639581368094	1.11351598949542	1.434725847386	0.000291284852147283
TSPAN32	0.786154698035708	0.694343832544996	0.890105420794624	0.000146290467385089
CASP14	1.09434484151395	1.04301037949847	1.1482058622696	0.000235190761741837
SLC13A5	1.13013854508598	1.05077443827152	1.21549695593093	0.000990762277181068
43894	1.14485372010087	1.05854609880675	1.23819835707323	0.00071776850366369
KRT16	1.10307522475115	1.04171895703574	1.16804531898142	0.000780198110019921
PAQR9	1.19623889829599	1.08319592195565	1.32107910747378	0.000403384647847666
CREG2	1.16173457987512	1.08235180466985	1.24693951472581	0.0000330470050230293
CDR1	1.20596997721884	1.08739303632101	1.33747737696922	0.000390331676046308
		**miRNAs**		
**id**	**HR**	**HR.95L**	**HR.95H**	**pvalue**
hsa-mir-6850	1.22369494655568	1.01328374596145	1.47779862076549	0.0359895941569773
hsa-mir-196b	1.08294261130757	1.01738752732888	1.15272171899405	0.0123833915312981
hsa-mir-548f-1	1.27222024842001	1.10341351793822	1.46685203160661	0.000916867328631251
hsa-mir-142	0.861346642636693	0.759325742488977	0.977074787889563	0.0203120234961398
hsa-mir-5571	0.817872466198366	0.703149376285542	0.951313324771772	0.00912789948313501

Supplementary Table 3. 88 immune DEGs.

Upregulated	Downregulated
IRF8	TENM1
LAIR1	IFNK
SIRPG	TNFSF11
MNDA	CALCA
PLA2G2D	SCG2
NFAM1	INHA
HLA-E	IL17C
SPI1	RETNLB
LY86	
CRTAM	
FOXP3	
FCGR3A	
CD27	
IL4I1	
HLA-DOA	
EVI2B	
TNFAIP8L2	
PTCRA	
HVCN1	
TLR8	
IL2RA	
CCL18	
CCL19	
TIGIT	
CYBB	
LST1	
CCL23	
CCR8	
RASGRP4	
CD244	
CXCL9	
CD83	
PRF1	
C5AR1	
FCN1	
TNFRSF9	
DCSTAMP	
CD79A	
GPR65	
HLA-DRB5	
CCRL2	
IL7R	
NCR1	
IL27	
CCL2	
TLR4	
CD19	
TPSAB1	

TNFRSF17
HAMP
CD68
CCL1
CD70
AIM2
IL34
IL21
CXCL13
CTLA4
CTSG
CX3CR1
AQP9
CCL7
LAG3
MMP9
CCL14
INS
FCAR
IL22RA2
VTN
IFNG
SPACA3
CCL25
CCL26
LTF
MMP7
CEACAM8
IL17A
EREG
IL1RL1
PYDC1
